# m^6^A‐Mediated Glycolysis by IL‐37 Drives T Cell Metabolic Reprogramming to Regulate Colitis

**DOI:** 10.1002/advs.202520472

**Published:** 2026-06-09

**Authors:** Xiaoyan Wang, Jiadong Yu, Guolin Li, Ya Li, Fanlian Zeng, Jing Hu, Yifan Zhou, Chengcheng Yue, Qixiang Zhao, Chen Zhang, Hong Zhou, Fulei Zhao, Yawen Hu, Pei Zhou, Linna Gu, Yuting Feng, Mingxiang He, Shishi Huang, Xiaochi Sun, Xinmeng Wang, Jie Tang, Xinyu Zeng, Wenling Wu, Nongyu Huang, Xinai Cui, Kaijun Cui, Jiong Li

**Affiliations:** ^1^ State Key Laboratory of Biotherapy and Cancer Center West China Hospital Sichuan University, and Collaborative Innovation Center for Biotherapy Chengdu Sichuan China; ^2^ Department of Cardiology West China Hospital Sichuan University Chengdu Sichuan China; ^3^ CDUTCM‐KEELE Joint Health and Medical Sciences Institute Chengdu University of Traditional Chinese Medicine Chengdu China

**Keywords:** colitis, glycolysis, IL‐37, m^6^A methylation, T cell metabolism

## Abstract

N6‐methyladenosine (m^6^A) modification and T‐cell metabolic reprogramming are increasingly recognized as critical drivers of inflammatory bowel disease (IBD). However, how the anti‐inflammatory cytokine interleukin‐37 intersects with m^6^A‐mediated metabolic regulation remains unclear. Here, we show that IL‐37 alleviates colitis by reducing global m^6^A levels and reshaping CD4^+^ T‐cell metabolism. Mechanistically, IL‐37 signals through its receptor SIGIRR to inhibit IRAK4 and JNK phosphorylation, suppress NF‐κB p65 activation, and downregulate METTL14, thereby decreasing m^6^A deposition. The IL‐37/METTL14 axis notably reduces m^6^A enrichment at the A2445 site in the 3′UTR of SLC2A1, destabilizing its mRNA and suppressing glycolysis. In vitro T‐cell polarization and adoptive transfer of METTL14‐overexpressing CD4^+^ T cells confirmed that this metabolic shift restrains Th1/Th17 differentiation while promoting Th2 expansion. Together, these findings reveal the IL‐37/SIGIRR–METTL14–m^6^A axis as a novel regulator of T‐cell metabolism and highlight SLC2A1 as a potential therapeutic target in IBD.

## Graphical abstract

This study identifies an IL‐37/SIGIRR‐METTL14 regulatory axis that suppresses global m^6^A modification in CD4+ T cells. IL‐37 signaling, mediated through SIGIRR, inhibits IRAK4 and JNK phosphorylation, leading to downregulation of the methyltransferase METTL14. Consequently, m^6^A methylation of GLUT1 mRNA is reduced, destabilizing the transcript and decreasing GLUT1 expression. The resulting impairment of glycolysis suppresses Th cell differentiation, revealing a novel immunometabolic mechanism with therapeutic potential for IBD.

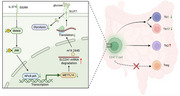

## 1 Introduction

N6‐methyladenosine (m^6^A) is one of the most prevalent post‐transcriptional modifications in eukaryotic mRNA, dynamically regulating RNA metabolism, including stability, splicing, and translation, and thereby influencing diverse physiological and pathological processes [1, 2, 3, 4, 5]. Emerging evidence highlights a pivotal role for m^6^A modification in T cell differentiation and functional regulation, particularly by modulating the post‐transcriptional control of metabolism‐related genes, thereby affecting T cell metabolic reprogramming [6, 7].

Increasingly, cellular metabolism is recognized not merely as an energy source but as a central regulator of immune cell fate and function [8, 9, 10]. Metabolic pathways such as glycolysis, oxidative phosphorylation, and fatty acid oxidation orchestrate immune cell activation, differentiation, and effector functions through epigenetic remodeling, redox modulation, and signaling cascades [8, 11, 12]. In various immune cell types, the activation of lineage‐specific promoters initiates differentiation programs closely linked to metabolic reprogramming. For example, glycolytic flux in macrophages drives pro‐inflammatory M1 polarization via HIF‐1α stabilization, whereas fatty acid β‐oxidation supports anti‐inflammatory M2 phenotypes [13]. Similarly, in T cells, mTOR‐mediated metabolic reprogramming critically dictates the balance between effector T cell proliferation and regulatory T cell suppressive capacity [14]. Despite these advances, the role of m^6^A modification in regulating immune cell differentiation and its impact on disease progression remains incompletely characterized.

Interleukin‐37 (IL‐37), a member of the IL‐1 cytokine family, exerts potent immunosuppressive effects [15, 16]. Elevated IL‐37 expression is observed in patients with rheumatoid arthritis, psoriasis, ulcerative colitis, and Crohn's disease, highlighting its critical role in inflammatory disorders, particularly inflammatory bowel disease (IBD) [17, 18, 19, 20, 21]. The immunoregulatory function of IL‐37 is primarily mediated through its receptor SIGIRR. Extracellular IL‐37 interacts with the cell surface receptor SIGIRR, suppressing IRAK4 phosphorylation and the activation of downstream signaling pathways, including NF‐κB and MAPK, thereby exerting anti‐inflammatory effects [22, 23].However, whether m^6^A ‐mediated epitranscriptomic regulation and metabolic reprogramming participate in IL‐37–mediated immune modulation remains unclear.

Here, we integrate single‐cell transcriptomic analyses from patients with Crohn's disease, rhinitis, and arthritis to reveal significant alterations in the expression of key m^6^A regulatory genes in CD4^+^ T cells compared to healthy controls. Mechanistically, we demonstrate that IL‐37 binding to SIGIRR inhibits the IRAK4/JNK/NF‐κB signaling axis, downregulates METTL14 expression, and reduces global m^6^A abundance in CD4^+^ T cells. This epitranscriptomic remodeling specifically targets the critical metabolic gene SLC2A1, removing m^6^A deposition at the A2445 site within its 3'UTR, thereby accelerating mRNA decay and suppressing glycolytic flux. Consequently, metabolically reprogrammed T cells exhibit impaired Th1/Th17 differentiation with enhanced Th2 functionality, ultimately attenuating inflammatory pathology. Our findings reveal that IL‐37 orchestrates T cell metabolic reprogramming via the SIGIRR/METTL14/m^6^A axis, uncoverring a mechanistic link between SIGIRR‐mediated signaling and epitranscriptomic regulation.

These results not only expand the conceptual framework of IL‐37 immunomodulation but also suggest a novel therapeutic paradigm targeting the interplay among epigenome, metabolism, and immune function in inflammatory diseases.

## 2 Results

### 2.1 IL‐37 Decreases m^6^A Methylation Levels in CD4^+^ T Cells

**FIGURE 1:**
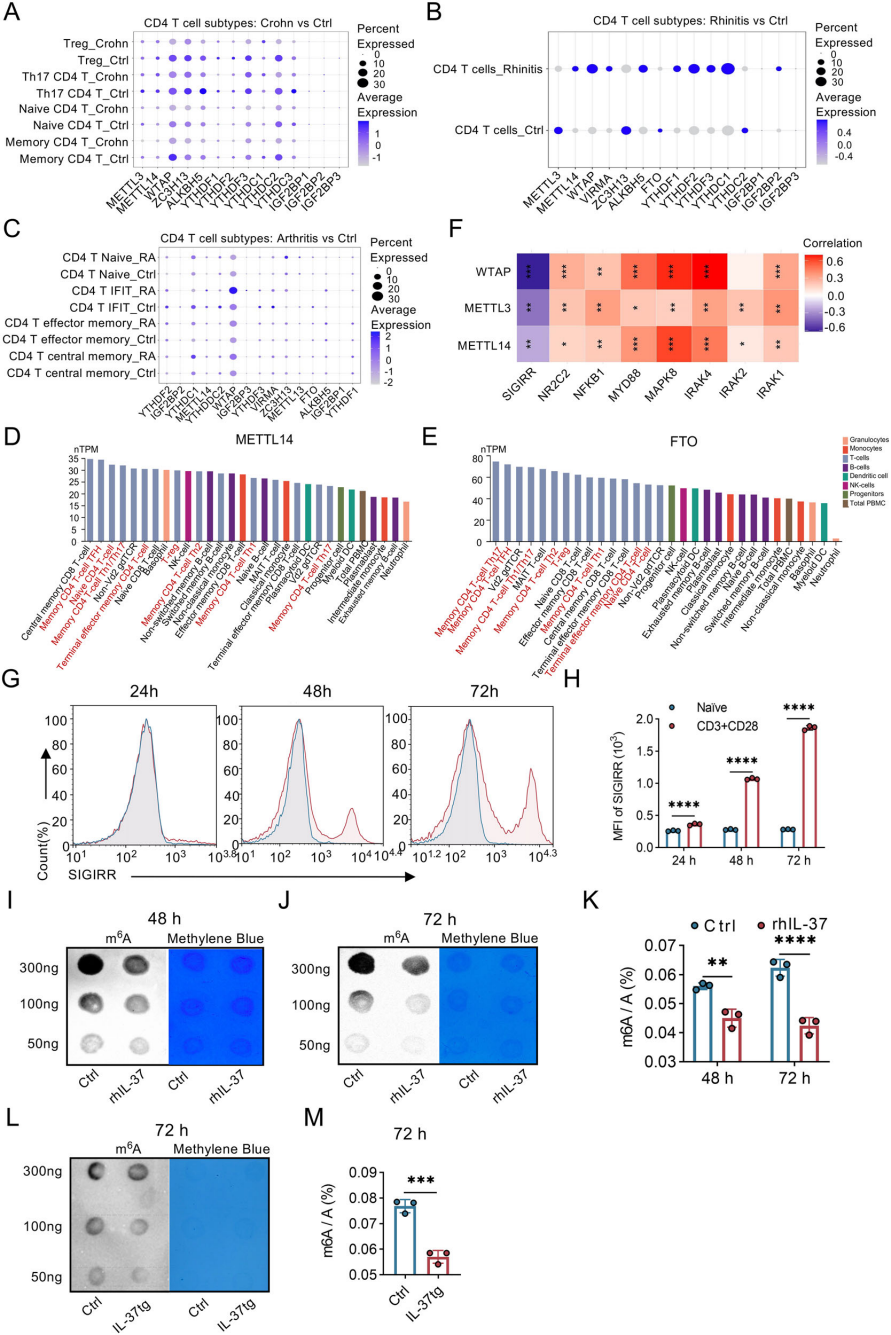
IL‐37 reduces the abundance of m^6^A methylation in CD4^+^T cells. (A) The dot plot shows the average expression of m^6^A methylation‐related genes in healthy individuals and Crohn's disease patients depicted using GEO datasets. (B) The dot plot shows the average expression of m^6^A methylation‐related genes in healthy individuals and rhinitis patients depicted using GEO datasets. (C) The dot plot shows the average expression of m^6^A methylation‐related genes in healthy individuals and arthritis patients depicted using Cellxgene discovery datasets. (D) The expression of METTL14 in different Human immune cells in The Human Protein Atlas database. (E) The expression of FTO in different Human immune cells in The Human Protein Atlas database. (F) Correlation between m^6^A “writer” genes and SIGIRR‐regulated signaling pathway genes in Crohn's disease pathogenesis based on GEO database analysis. Correlations between variables were assessed by linear regression analysis. Linear correction index R and p values were calculated. (G) Flow cytometry was used to detect the expression of SIGIRR in CD4^+^T cells after CD3/CD28 stimulation at 24, 48 and 72 h. (H) Average fluorescence intensity statistics of SIGIRR on CD4^+^T cells stimulated by CD3/CD28 at 24, 48, and 72 h (*n* = 3). (I,J) Spot hybridization was used to detect the m^6^A methylation abundance of CD4^+^T cells after CD3/CD28 activation for 48, and 72 h. (K) ELISA was used to quantitatively detect the m^6^A methylation abundance of CD4^+^T cells 48h, 72h after CD3/CD28 activation (*n* = 3). (L) Detection of m^6^A methylation abundance in CD4^+^ T cells from WT and IL‐37tg mice after 72 h of CD3/CD28 activation using dot blot assay. (M) ELISA was used to detect the m^6^A methylation abundance in CD4^+^ T cells from WT and IL‐37tg mice after 72 h of CD3/CD28 activation. Error bars represent the mean ± SEM. n=3 biologically independent experiments. ns, not significant; ^**^ *p* < 0.01; ^***^ *p* < 0.001 ^****^ *p* <0.0001; p values were calculated using Student's t test.

Accumulating evidence indicates that m^6^A modification plays a pivotal role in regulating CD4^+^ T cell function. To systematically characterize m^6^A regulatory patterns in immune‐related diseases, we integrated single‐cell RNA‐seq datasets from the GEO database (GSE157477 and GSE179265) and CellxGene Discover (ID0543692) [24, 25, 26]. Analysis of Crohn's disease, chronic rhinitis, and arthritis—diseases characterized by CD4^+^ T cell dysregulation—revealed distinct expression patterns of m^6^A writer, eraser, and reader genes in CD4^+^ T cell subsets. Specifically, METTL3, METTL14, WTAP, and ALKBH5 were differentially expressed in Crohn's disease (Figure 1A), while METTL3, METTL14, WTAP, ALKBH5, and FTO were altered in chronic rhinitis (Figure 1B). In arthritis, WTAP and ALKBH5 showed significant changes (Figure 1C). These findings indicate that key m^6^A regulatory proteins are consistently dysregulated across multiple immune‐related diseases, suggesting a potential role for m^6^A‐mediated epitranscriptomic regulation in pathological immune responses.

Further analysis using the Human Protein Atlas (HPA) database showed that METTL14 and FTO are highly expressed in CD4^+^ T cells (Figure 1D,E), whereas METTL3, WTAP, and ALKBH5 exhibit cell‐type‐specific enrichment (Figure S1A–C), supporting a potential functional role for METTL14 and FTO in CD4^+^ T cell regulation. Given the observed dysregulation of m^6^A regulators, we next sought to identify upstream signaling pathways that may control m^6^A modification in CD4^+^ T cells. IL‐37, a key anti‐inflammatory cytokine that signals through its receptor SIGIRR, is known to suppress inflammatory responses mediated by Toll‐like receptors and IL‐1 receptor family members. Analysis of an inflammatory bowel disease dataset (GSE279302) revealed that SIGIRR expression was negatively correlated with core m^6^A methyltransferases (METTL3, METTL14, and WTAP), while pro‐inflammatory genes showed positive correlations with these methyltransferases (Figure 1F). These findings suggest that IL‐37–SIGIRR signaling may act as an upstream regulatory axis that suppresses m^6^A methylation in CD4^+^ T cells, providing a rationale for further investigation of IL‐37–mediated epitranscriptomic regulation.

To further define the role of IL‐37 in CD4^+^ T cell function, we examined its effects using an in vitro TCR‐dependent differentiation system [27, 28]. rhIL‐37 treatment significantly reduced Th1 and Th17 differentiation while promoting Th2 and Treg differentiation (Figure S2A,B). Flow cytometry analysis further showed that IL‐37 did not affect CD4^+^ T cell proliferation or apoptosis (Figure S2C–F), indicating that IL‐37 selectively regulates T cell differentiation without altering cell survival. In a T cell adoptive transfer colitis model, IL‐37–mediated changes in CD4^+^ T cell differentiation were associated with reduced intestinal inflammation, confirming its protective role in inflammatory bowel disease (Figure S3). We next investigated whether IL‐37 regulates CD4^+^ T cell function through m^6^A modification. In naïve CD4^+^ T cells, no significant changes in m^6^A abundance were observed following IL‐37 treatment at 24, 48, or 72 h (Figure S4A–C), suggesting that IL‐37 does not affect basal methylation levels. TCR signaling is indispensable for T cell activation, proliferation, and differentiation [29]. Notably, SIGIRR expression increased over time following TCR stimulation (Figure 1G,H), indicating that IL‐37 signaling may be preferentially engaged during T cell activation. Consistent with this, dot blot and ELISA analyses showed that IL‐37 significantly reduced m^6^A levels in activated CD4^+^ T cells at 48 h and 72 h (Figure 1I–K). Similarly, CD4^+^ T cells from IL‐37tg mice exhibited lower m^6^A levels compared with WT controls following activation (Figure 1L,M). Collectively, these findings suggest that IL‐37 regulates CD4^+^ T cell differentiation by reducing m^6^A modification during T cell activation, thereby contributing to the suppression of CD4^+^ T cell–mediated inflammatory responses.

### 2.2 IL‐37 Regulates CD4^+^ T Cell Differentiation by Downregulating METTL14 via the SIGIRR‐Mediated IRAK4/JNK/NF‐κB Pathway

**FIGURE 2:**
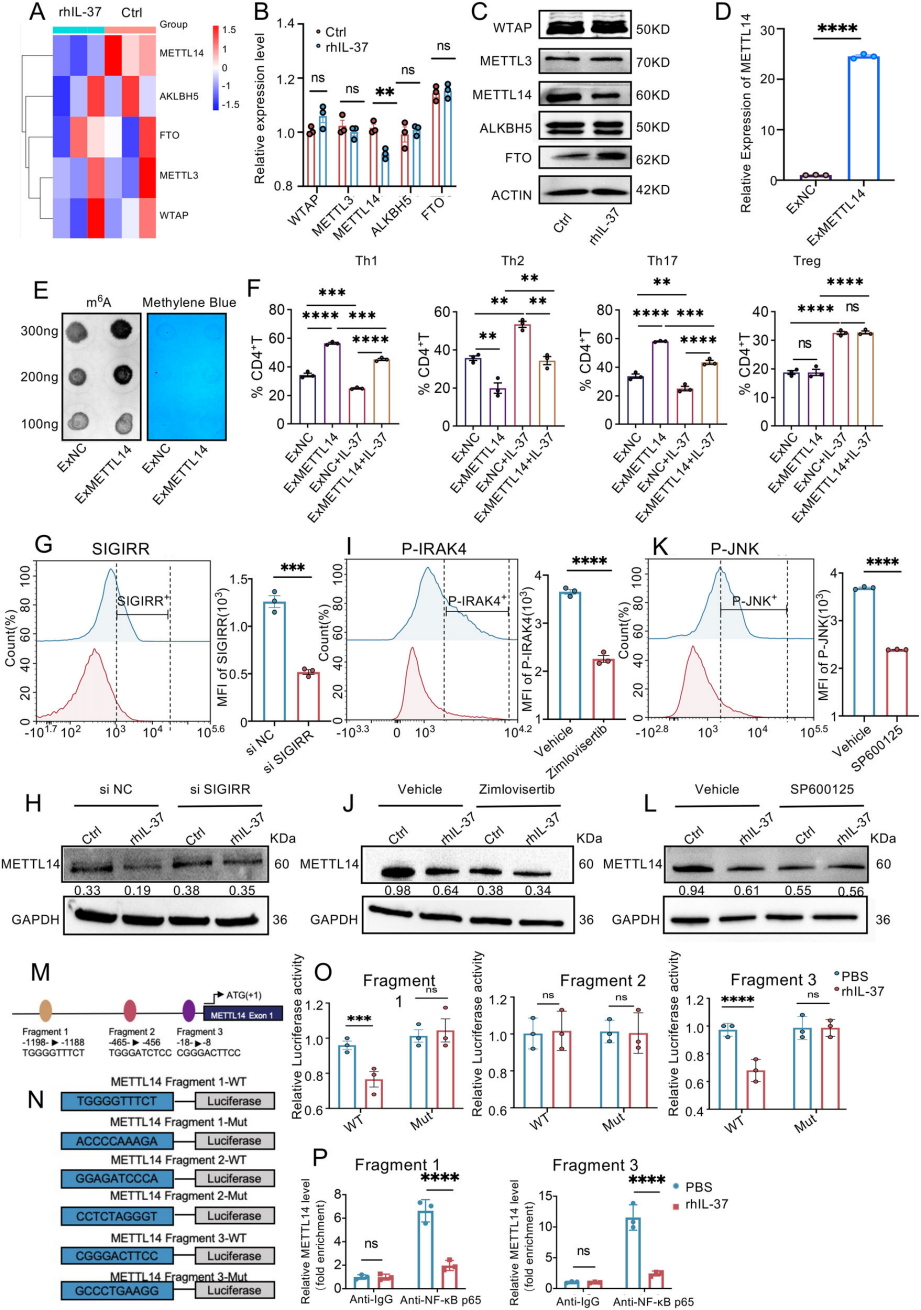
IL‐37 inhibits the phosphorylation of IRAK4 and JNK downstream of SIGIRR and down‐regulates the expression of METTL14 by suppressing the activity of NF‐κB P65, thereby regulating the differentiation of CD4^+^ T cells. (A) The heatmap shows the differential expression of METTL3, METTL14, WTAP, FTO and ALKBH5 mRNA in RNA‐seq data of CD4^+^ T cells with and without IL‐37 (*n* = 3). (B) The mRNA expression levels of METTL3, METTL14, WTAP, FTO, and ALKBH5 in CD4^+^ T cells with and without IL‐37 were detected by RT‐qPCR (*n* = 3). (C) The protein expression levels of METTL3, METTL14, WTAP, FTO, and ALKBH5 in CD4^+^ T cells with and without IL‐37 were detected by WB. (D) The expression of METTL14 in CD4^+^ T cells after METTL14 overexpression virus infection was detected by RT‐qPCR (*n* = 3). (E) The m^6^A methylation abundance in CD4^+^ T cells after METTL14 overexpression virus infection was detected by dot blotting. (F) Flow cytometry shows that IL‐37 can counteract the differentiation shift of CD4^+^ T cell subsets Th1, Th2, Th17, and Treg caused by METTL14 overexpression (*n* = 3). (G) Flow cytometry was used to detect the interference effect of siRNA on SIGIRR expression in CD4^+^ T cells (left). The average fluorescence intensity of SIGIRR (right, *n* = 3). (H) The effect of IL‐37 on METTL14 expression in CD4^+^ T cells after SIGIRR knockdown was examined by Western blotting. (I) Flow cytometry was used to detect the inhibitory effect of P‐IRAK4 inhibitor on P‐IRAK4 expression in CD4^+^ T cells (left). The average fluorescence intensity of P‐IRAK4 (right, *n* = 3). (J) Western blot analysis of METTL14 expression in CD4^+^ T cells treated with IL‐37 after P‐IRAK4 inhibition. (K) Flow cytometry was used to detect the inhibitory effect of P‐JNK inhibitor on P‐JNK expression in CD4^+^ T cells (left). The average fluorescence intensity of P‐JNK (right, *n* = 3). (L) Western blot analysis of METTL14 expression in CD4^+^ T cells treated with IL‐37 after P‐JNK inhibition. (M) JASPAR online website was used to predict the potential binding Fragment of NF‐κB p65 and METTL14 gene promoter regions. (N) The potential binding Fragment of NF‐κB p65 and METTL14 gene promoter regions and their mutant sequences. (O) The fluorescence intensities of the wild‐type and mutant at Fragment 1, Fragment 2, and Fragment 3 after adding PBS and rhIL‐37 (*n* = 3). (p) Chromatin immunoprecipitation (ChIP) assay was performed to detect the binding of NF‐κB p65 to two fragments of the METTL14 gene. IgG was used as a negative control(*n* = 3). Error bars represent the mean ± SEM. *n* = 3 biologically independent experiments. ^*^ *p* < 0.05; ^**^ *p* < 0.01; ^***^ *p* < 0.001; ^****^ *p* <0.0001; p values were calculated using Student's t test or One‐way ANOVA and Two‐way ANOVA.

To elucidate the mechanism by which IL‐37 reduces m^6^A methylation in CD4^+^ T cells, we analyzed the expression of core m^6^A regulators. RNA sequencing revealed that METTL14 mRNA was significantly downregulated following rhIL‐37 treatment, whereas WTAP, METTL3, ALKBH5, and FTO remained unchanged (Figure 2A). RT‐qPCR results were consistent with these findings (Figure 2B). Western blot analysis further confirmed that IL‐37 suppressed METTL14 protein expression and increased FTO protein levels (Figure 2C). To determine whether METTL14 mediates IL‐37–induced effects, METTL14 was overexpressed in CD4^+^ T cells using an adenoviral system (Figure 2D), which increased global m^6^A abundance (Figure 2E). In a TCR‐dependent differentiation system, METTL14 overexpression promoted Th1 and Th17 differentiation, reduced Th2 differentiation, and had no effect on Treg cells. Notably, rhIL‐37 treatment reversed these changes (Figure 2F). Consistently, METTL14 silencing decreased Th1 and Th17 differentiation while increasing Th2 differentiation, without affecting Treg cells. Importantly, rhIL‐37 failed to modulate T helper cell differentiation under METTL14‐deficient conditions (Figure S5), indicating that METTL14 is required for IL‐37–mediated immunoregulation. We next evaluated the role of FTO using the specific inhibitor Dac51. FTO inhibition did not significantly affect CD4^+^ T cell differentiation (Figure S6A,B) or IL‐37–mediated effects (Figure S6C). Similarly, siRNA‐mediated FTO silencing produced comparable results (Figure S6D,E). Previous studies also reported that FTO expression remains unchanged during Th cell differentiation and CD3/CD28 activation, suggesting a limited role in CD4^+^ T cell function [30]. Together, these results identify METTL14, but not FTO, as a key mediator of IL‐37–regulated CD4^+^ T cell differentiation. Therefore, we next focused on the mechanism by which IL‐37 suppresses METTL14 expression.

Since IL‐37 exerts its effects through SIGIRR, we first examined whether SIGIRR mediates METTL14 regulation. SIGIRR was silenced using siRNA, and METTL14 protein levels were assessed by Western blot. IL‐37 failed to downregulate METTL14 following SIGIRR knockdown (Figure 2G,H), indicating that this effect is SIGIRR‐dependent.We next investigated the downstream signaling pathways. Treatment with Zimlovisertib (IRAK4 phosphorylation inhibitor) or SP600125 (JNK phosphorylation inhibitor) abrogated IL‐37–mediated regulation of METTL14 (Figure 2I–L), indicating that IRAK4 and JNK signaling are involved. Given that NF‐κB p65 is a key downstream transcription factor of SIGIRR signaling [31, 32], we examined its potential role in regulating METTL14 transcription. JASPAR analysis (https://jaspar.elixir.no) identified three putative binding regions in the METTL14 promoter (Figure 2M). Dual‐luciferase reporter assays showed that IL‐37 significantly altered the activity of fragments 1 and 3, but not fragment 2 (Figure 2N,O). ChIP‐qPCR analysis further demonstrated that NF‐κB p65 binding to fragments 1 and 3 was significantly enriched under basal conditions and markedly reduced following IL‐37 treatment (Figure 2P), indicating that IL‐37 suppresses NF‐κB p65 binding to the METTL14 promoter. Collectively, these findings demonstrate that IL‐37 downregulates METTL14 via the SIGIRR–IRAK4/JNK–NF‐κB signaling pathway.

### 2.3 IL‐37 Attenuates METTL14 Overexpression–Exacerbated Adoptive Transfer Colitis

**FIGURE 3:**
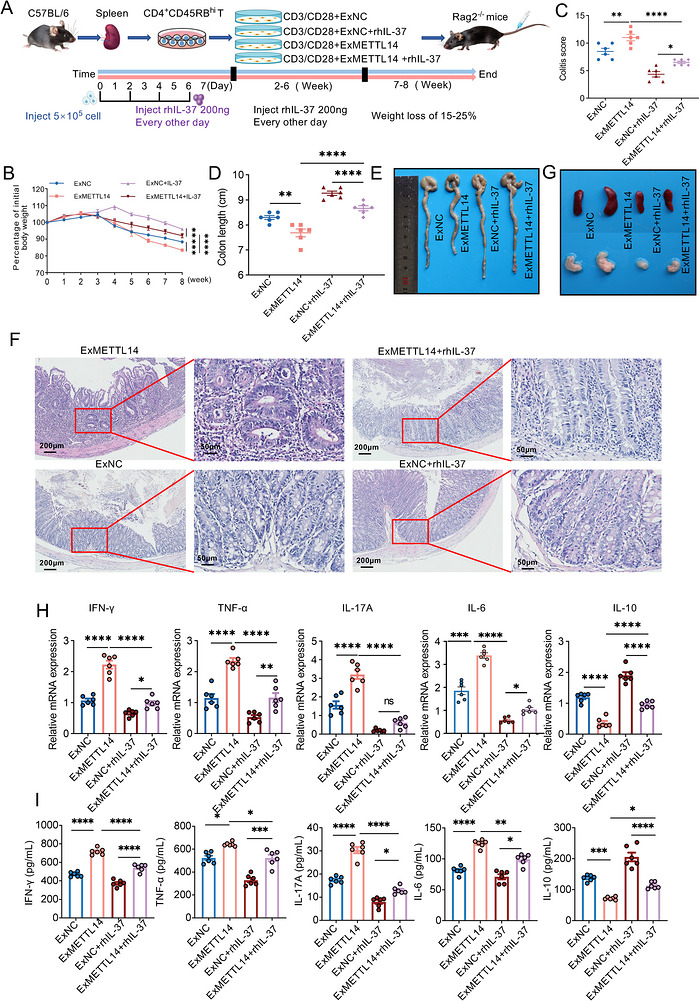
IL‐37 alleviates adoptive transfer colitis exacerbated by promoting METTL14 overexpression. (A) Schematic diagram of the adoptive transfer colitis model in Rag2^−/−^ mice by reinfusing control CD4^+^ T cells, CD4^+^ T cells overexpressing METTL14, IL‐37‐treated control CD4^+^ T cells, and IL‐37‐treated CD4^+^ T cells overexpressing METTL14. (B) Body weight changes during the development of adoptive transfer colitis after reinfusing control CD4^+^ T cells, CD4^+^ T cells overexpressing METTL14, IL‐37‐treated control CD4^+^ T cells, and IL‐37‐treated CD4^+^ T cells overexpressing METTL14 into Rag2^−/−^ mice (n=6). (C) Disease severity scores of mice after reinfusing control CD4^+^ T cells, CD4^+^ T cells overexpressing METTL14, IL‐37‐treated control CD4^+^ T cells, and IL‐37‐treated CD4^+^ T cells overexpressing METTL14 into Rag2^−/−^ mice for 8 weeks (*n* = 6). (D) Statistical graph of colon length of mice after reinfusing control CD4^+^ T cells, CD4^+^ T cells overexpressing METTL14, IL‐37‐treated control CD4^+^ T cells, and IL‐37‐treated CD4^+^ T cells overexpressing METTL14 into Rag2^−/−^ mice for 8 weeks (*n* = 6). (E) Representative images of the colon of mice after reinfusing control CD4^+^ T cells, CD4^+^ T cells overexpressing METTL14, IL‐37‐treated control CD4^+^ T cells, and IL‐37‐treated CD4^+^ T cells overexpressing METTL14 into Rag2^−/−^ mice for 8 weeks. (F) HE staining of colon tissue of mice after reinfusing control CD4^+^ T cells, CD4^+^ T cells overexpressing METTL14, IL‐37‐treated control CD4^+^ T cells, and IL‐37‐treated CD4^+^ T cells overexpressing METTL14 into Rag2^−/−^ mice for 8 weeks (scale bar = 200 or 50µm). (G) Schematic diagram of the size of the spleen and mesenteric lymph nodes of mice after reinfusing control CD4^+^ T cells, CD4^+^ T cells overexpressing METTL14, IL‐37‐treated control CD4^+^ T cells, and IL‐37‐treated CD4^+^ T cells overexpressing METTL14 into Rag2^−/−^ mice for 8 weeks. (H) RT‐qPCR detection of mRNA expression of inflammatory factors IFN‐γ, IL‐10, IL‐17A, IL‐6, and TNF‐α in colon tissue of mice after reinfusing control CD4^+^ T cells, CD4^+^ T cells overexpressing METTL14, IL‐37‐treated control CD4^+^ T cells, and IL‐37‐treated CD4^+^ T cells overexpressing METTL14 into Rag2^−/−^ mice for 8 weeks (*n* = 6). (I) ELISA was used to detect the protein levels of IFN‐γ, TNF‐α, IL‐6, IL‐17A and IL‐10 in the serum of Rag2^−/−^ mice 8 weeks after the infusion of control CD4^+^ T cells, CD4^+^ T cells overexpressing METTL14, control CD4^+^ T cells treated with IL‐37, and CD4^+^ T cells overexpressing METTL14 treated with IL‐37 (*n* = 6). Error bars represent the mean ± SEM. *n* = 6 biologically independent mice per group. ^*^ *p* < 0.05; ^**^ *p* < 0.01; ^***^ *p* < 0.001; ^****^ *p* <0.0001; p values were calculated using Student's t test or One‐way ANOVA and Two‐way ANOVA.

Inflammatory bowel disease (IBD) is a chronic and relapsing intestinal disorder characterized by CD4^+^ T cell dysfunction, with clinical manifestations including diarrhea, abdominal pain, and bloody stools [33, 34]. To investigate whether IL‐37 regulates CD4^+^ T cell differentiation via METTL14 in vivo, we established an adoptive T cell transfer model using Rag2^−^/^−^ mice (Figure 3A). Four groups of activated CD4^+^ T cells were transferred: control, METTL14‐overexpressing, rhIL‐37–treated control, and rhIL‐37–treated METTL14‐overexpressing cells. Mice receiving METTL14‐overexpressing CD4^+^ T cells exhibited significant weight loss starting three weeks post‐transfer, which was partially reversed by rhIL‐37 treatment (Figure 3B). Consistently, METTL14 overexpression increased colitis severity scores, while rhIL‐37 reduced disease severity (Figure 3C). Colon length measurements further supported these findings (Figure 3D,E). Histological analysis revealed severe colonic damage in mice receiving METTL14‐overexpressing CD4^+^ T cells, including crypt loss, epithelial injury, goblet cell depletion, and inflammatory infiltration, whereas rhIL‐37 treatment markedly alleviated these pathological changes (Figure 3F). In addition, spleen and mesenteric lymph node enlargement were consistent with disease severity and were reduced by rhIL‐37 treatment (Figure 3G). These results demonstrate that METTL14 overexpression exacerbates IBD pathology, whereas IL‐37 partially reverses this effect.

To evaluate inflammatory responses, cytokine levels were measured in colonic tissues and serum. Compared with mice receiving control CD4^+^ T cells, METTL14 overexpression significantly increased the expression of pro‐inflammatory cytokines IFN‐γ, TNF‐α, IL‐6, and IL‐17A, while reducing IL‐10 levels in colonic tissues. These changes were reversed by rhIL‐37 treatment (Figure 3H). ELISA analysis of serum cytokines showed a similar trend (Figure 3I). These results indicate that METTL14 promotes inflammatory responses in adoptive transfer colitis, whereas IL‐37 effectively counteracts this effect.

In the DSS‐induced murine colitis model, immunofluorescence analysis showed that METTL14 expression in colonic CD4^+^ T cells was significantly reduced in IL‐37tg mice compared with WT controls (Figure S7A), further supporting the involvement of METTL14 in intestinal inflammation.

### 2.4 IL‐37 Modulates CD4^+^ T Cell Function by Regulating m^6^A Methylation in Metabolic Signaling Pathways

**FIGURE 4:**
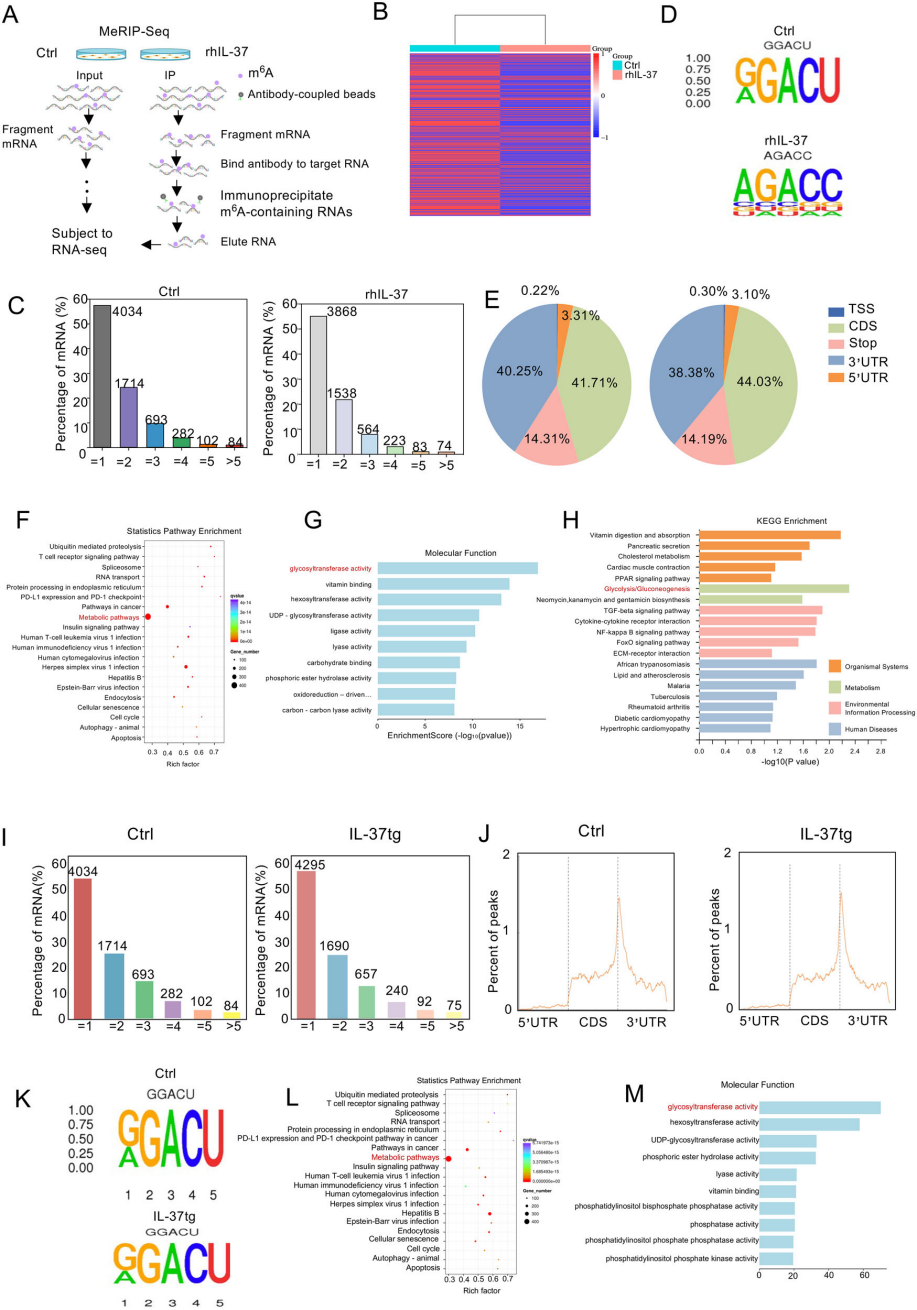
IL‐37 regulates the function of CD4^+^ T cells by altering the enrichment of m^6^A in metabolic signaling pathways. (A) Schematic diagram of the MeRIP‐seq principle for CD4^+^ T cells with or without IL‐37 treatment. (B) Heatmap of MeRIP‐seq analysis of CD4^+^ T cells with or without IL‐37 treatment. (C) Number of mRNA genes with different numbers of m^6^A peaks in sequencing data of CD4^+^ T cells with or without IL‐37 treatment. (D) Consensus motif map of MeRIP‐seq peaks identified in CD4^+^ T cells with or without IL‐37 treatment. (E) Percentage of m^6^A peaks in different regions of mRNA in non‐overlapping transcript fragments. (F) GO classification of m^6^A‐enriched differential genes in CD4^+^ T cells with or without IL‐37 treatment. (G) GO classification analysis of metabolic signaling pathway genes with differential m^6^A peaks identified by MeRIP‐seq in CD4^+^ T cells with or without IL‐37 treatment. (H) KEGG analysis of differential genes after RNA‐seq in CD4^+^ T cells with or without IL‐37 treatment. (I) The number of mRNA genes with different numbers of m^6^A peaks in the MeRIP‐seq data of CD4^+^T cells from IL‐37tg mice and WT mice. (J)Distribution of fold changes in m^6^A peak enrichment across different mRNA regions in CD4^+^ T cells from WT and IL‐37tg mice. (K) The consensus motif map of peaks in the MeRIP‐seq data of CD4^+^T cells from IL‐37tg mice and WT mice. (L) GO classification analysis of m^6^A‐enriched differentially expressed genes in CD4^+^T cells from IL‐37tg mice and WT mice. (M) GO classification analysis of metabolic signaling pathway genes with differential m^6^A peaks identified by MeRIP‐seq in CD4^+^ T cells from WT and IL‐37tg mice.

To investigate the regulatory effects of IL‐37 on m^6^A modification in CD4^+^ T cells, we performed MeRIP‐seq analysis (Figure 4A). Differential m^6^A modification patterns were observed following rhIL‐37 treatment, with both increased and decreased peaks (Figure 4B). Overall, IL‐37 reduced the number of m^6^A peaks across transcripts (Figure 4C), consistent with previous dot blot assays and ELISA measurements showing decreased global m^6^A levels. m^6^A is typically enriched within the 5′‐DRACH consensus sequence (D = G/A/U, R = A/G, H = non‐G). Motif analysis revealed that m^6^A peaks in both groups were primarily enriched in the RRACH consensus sequence (R = G or A, H = A, C, or U) (Figure 4D). The distribution of m^6^A across different transcript regions, such as coding sequences (CDS) and untranslated regions (UTRs), is associated with distinct biological functions and regulatory mechanisms. In CD4^+^ T cells, m^6^A peaks were mainly distributed in the CDS, 3′ UTR, and stop codon regions. IL‐37 treatment increased m^6^A enrichment in the CDS region while decreasing enrichment in the 3′ UTR (Figure 4E), suggesting that IL‐37 may influence gene expression by modulating the regional distribution of m^6^A. Gene Ontology (GO) analysis showed that differentially methylated genes were mainly associated with cellular metabolic processes (Figure 4F), with significant enrichment in molecular functions related to glycosyltransferase activity (Figure 4G). To further explore the impact of IL‐37–mediated m^6^A regulation on gene expression, RNA‐seq analysis was performed. KEGG pathway analysis revealed that IL‐37 significantly affected multiple inflammation‐related signaling pathways, including TGF‐β and NF‐κB, as well as metabolic pathways. Notably, the glycolysis–gluconeogenesis pathway showed the most significant enrichment (Figure 4H). Glycolysis–gluconeogenesis is a central metabolic process responsible for glucose catabolism and synthesis and is essential for maintaining cellular energy homeostasis and biosynthesis [35]. Consistently, MeRIP‐seq analysis of CD4^+^ T cells from wild‐type and IL‐37 transgenic mice showed reduced m^6^A methylation levels in IL‐37 transgenic mice, with differentially methylated genes significantly enriched in metabolic pathways (Figure 4I–M). Collectively, these results indicate that IL‐37 regulates m^6^A modification in CD4^+^ T cells and is associated with metabolic pathway remodeling.

### 2.5 IL‐37 Inhibits SLC2A1 Expression by Reducing mRNA Stability in a METTL14‐Dependent Manner

**FIGURE 5:**
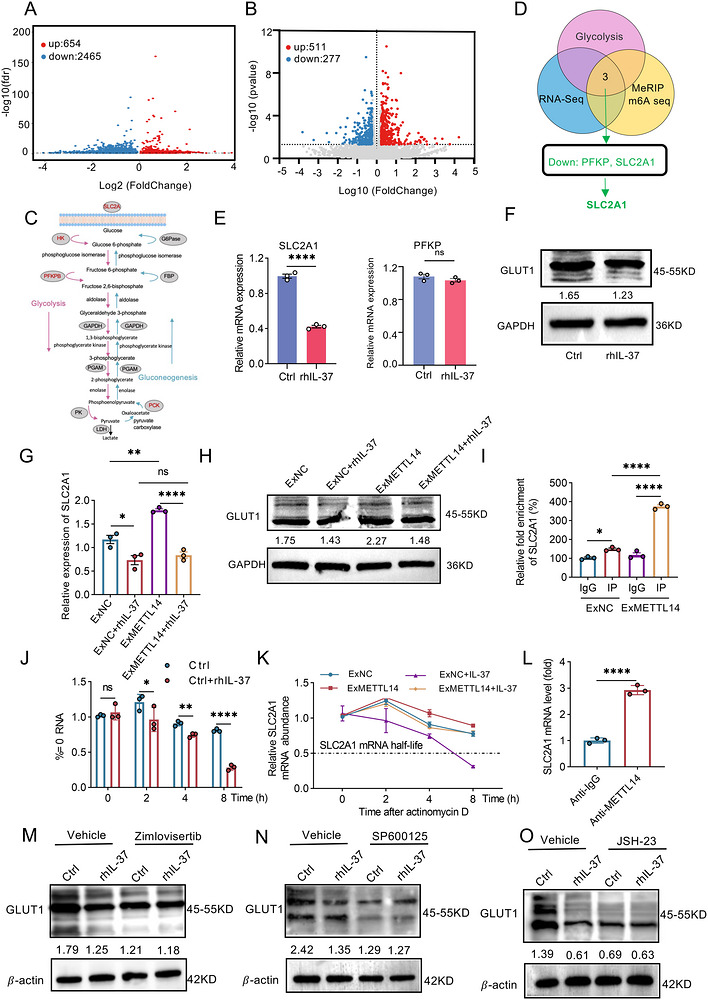
IL‐37 destabilizes SLC2A1 mRNA in a METTL14‐dependent manner. (A) Volcano plot of genes with differential m^6^A peaks in CD4^+^ T cells with or without IL‐37 treatment. (B) Volcano plot of differentially expressed genes in CD4^+^ T cells with or without IL‐37 treatment. (C) Genes that are differentially in RNA‐seq and MeRIP‐seq of CD4^+^ T cells with or without IL‐37 treatment are marked in the glycolysis process. (D) Workflow for identifying downstream target genes of IL‐37. (E) RT‐qPCR detection of mRNA expression levels of the downstream gene PFKP and SLC2A1 screened out (*n* = 3). (F) The expression levels of SLC2A1 by WB. (G) The expression levels of SLC2A1 mRNA in control CD4^+^ T cells, CD4^+^ T cells treated with IL‐37, CD4^+^ T cells overexpressing METTL14, and CD4^+^ T cells overexpressing METTL14 treated with IL‐37 were detected by RT‐qPCR (*n* = 3). (H)The expression levels of SLC2A1 protein in control CD4^+^ T cells, CD4^+^ T cells treated with IL‐37, CD4^+^ T cells overexpressing METTL14, and CD4^+^ T cells overexpressing METTL14 treated with IL‐37 were detected by WB. (I) RIP‐qPCR was used to detect the expression of SLC2A1 mRNA in control CD4^+^ T cells and CD4^+^ T cells overexpressing METTL14 using an m^6^A antibody, with rabbit IgG as a control (*n* = 3). (J) The expression levels of SLC2A1 mRNA in control CD4^+^ T cells and CD4^+^ T cells treated with IL‐37 at 0, 2, 4, and 8 h after actinomycin D treatment (*n* = 3). (K) The expression levels of SLC2A1 mRNA in control CD4^+^ T cells, CD4^+^ T cells treated with IL‐37, CD4^+^ T cells overexpressing METTL14, and CD4^+^ T cells overexpressing METTL14 treated with IL‐37 at 0, 2, 4, and 8 h after actinomycin D treatment (*n* = 3). (L) RIP‐qPCR was used to detect the expression of SLC2A1 mRNA in CD4^+^ T cells using a METTL14 antibody, with rabbit IgG as a control (*n* = 3). (M) Western blot analysis of GLUT1 expression in CD4^+^ T cells treated with IL‐37 after P‐IRAK4 inhibition. (N) Western blot analysis of GLUT1 expression in CD4^+^ T cells treated with IL‐37 after P‐JNK inhibition. (O) Western blot analysis of GLUT1 expression in CD4^+^ T cells treated with IL‐37 after NF‐κB p65 inhibition. *n* = 3 biologically independent experiments. Error bars represent the mean ± SEM. ^*^ *p* < 0.05; ^**^ *p* < 0.01; ^***^ *p* < 0.001; ^****^ *p* <0.0001; p values were calculated using Student's t test or One‐way ANOVA and Two‐way ANOVA.

To identify downstream targets of IL‐37–mediated m^6^A regulation, we integrated MeRIP‐seq and RNA‐seq analyses. MeRIP‐seq identified 654 genes with increased m^6^A peaks and 2465 genes with decreased peaks, while RNA‐seq revealed 511 upregulated and 277 downregulated genes (Figure 5A,B). Genes exhibiting both reduced m^6^A modification and decreased expression were primarily enriched in the glycolysis pathway (Figure 5C), suggesting that IL‐37 may regulate CD4^+^ T cell function through metabolic reprogramming. Based on KEGG analysis, SLC2A1 and PFKP were selected as candidate targets (Figure 5D). RT‐qPCR analysis showed that IL‐37 significantly reduced SLC2A1 mRNA levels, whereas PFKP expression remained unchanged. Consistently, IL‐37 markedly decreased SLC2A1 protein expression (Figure 5E,F), indicating that SLC2A1 may serve as a key downstream target of IL‐37. METTL14 overexpression increased SLC2A1 mRNA and protein levels, while IL‐37 reversed these effects (Figure 5G,H), suggesting that SLC2A1 expression is regulated in a METTL14‐dependent manner. m^6^A RIP‐qPCR further demonstrated that METTL14 overexpression significantly enhanced m^6^A enrichment on SLC2A1 mRNA (Figure 5I), supporting its role in m^6^A modification. Actinomycin D assays showed that IL‐37 accelerated SLC2A1 mRNA degradation (Figure 5J), whereas METTL14 overexpression increased mRNA stability, which was reversed by IL‐37 (Figure 5K), suggesting that IL‐37 may regulate SLC2A1 expression by modulating mRNA stability. METTL14 RIP‐qPCR further confirmed that METTL14 directly binds to SLC2A1 mRNA (Figure 5L), supporting a direct regulatory interaction Pharmacological inhibition of IRAK4 (Zimlovisertib) or JNK (SP600125) abolished IL‐37–mediated downregulation of SLC2A1 expression (Figure 5M,N). Similarly, inhibition of NF‐κB p65 (JSH‐23) reversed the suppressive effect of IL‐37 on SLC2A1 (Figure 5O), suggesting that SLC2A1 may be regulated through the IRAK4/JNK/NF‐κB signaling pathway.

In the DSS‐induced murine colitis model, immunofluorescence analysis showed that SLC2A1 expression in colonic CD4^+^ T cells was significantly reduced in IL‐37tg mice compared with WT controls (Figure S7B), further supporting its in vivo relevance. Collectively, these results indicate that IL‐37 suppresses SLC2A1 expression in a METTL14‐dependent manner by reducing mRNA stability and may thereby contribute to metabolic and immune regulation.

### 2.6 IL‐37–Mediated Downregulation of SLC2A1 Suppresses CD4^+^ T Cell Glycolysis to Modulate Th Cell Differentiation

**FIGURE 6:**
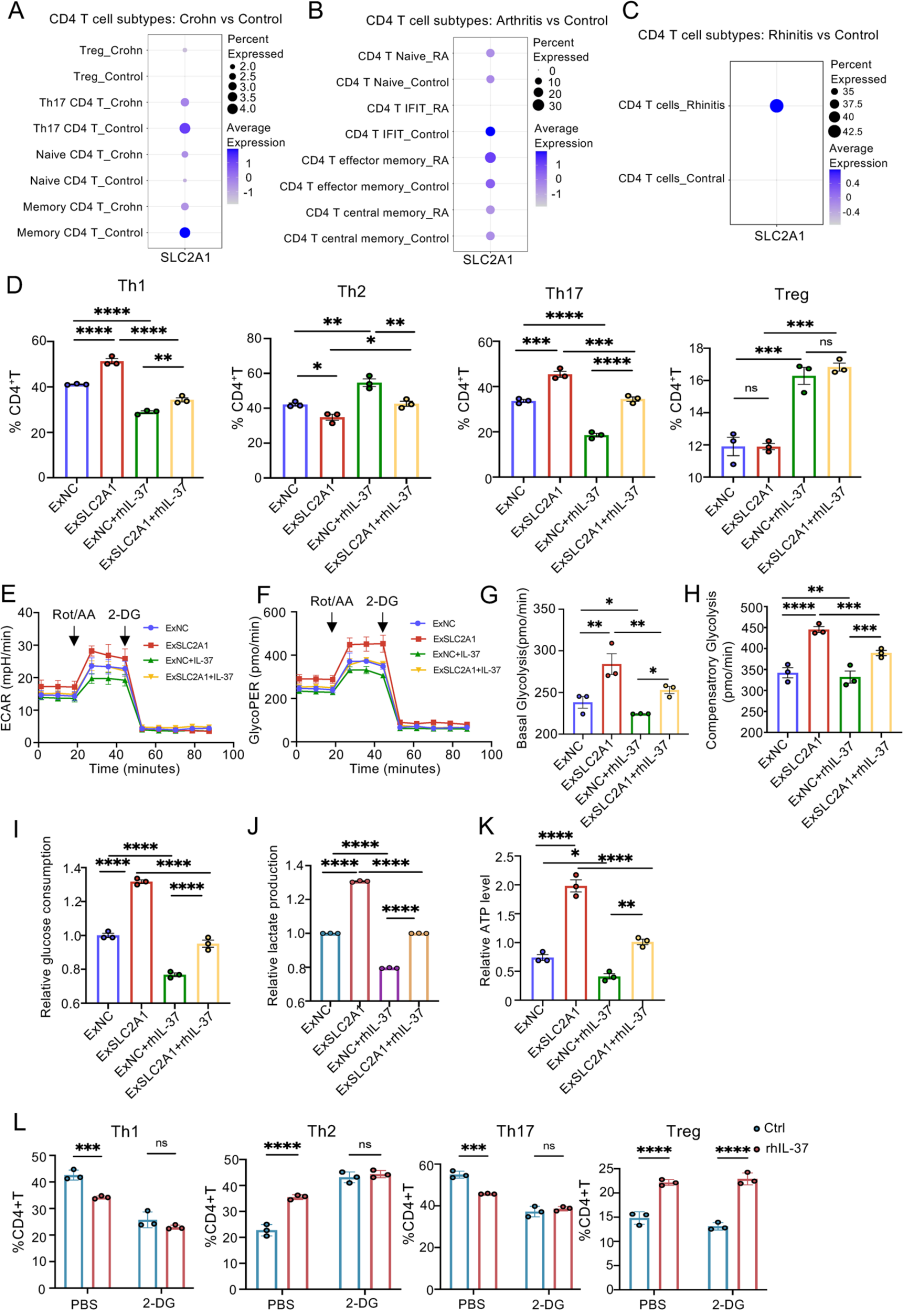
IL‐37 regulates Th cell differentiation by downregulating SLC2A1 and suppressing glycolysis in CD4^+^ T cells. (A) The dot plot shows the average expression of SLC2A1in healthy individuals and Crohn's disease patients depicted using published datasets. (B) The dot plot shows the average expression of SLC2A1 in healthy individuals and arthritis patients depicted using published datasets. (C) The dot plot shows the average expression of SLC2A1 in healthy individuals and rhinitis patients depicted using published datasets. (D) Flow cytometry was used to detect the differentiation of CD4^+^ T cells in four groups (control cells, SLC2A1 overexpression cells, IL‐37 treated cells, and IL‐37 treated SLC2A1 overexpression cells) (*n* = 3). (E–H) ECAR, glycolytic capacity (glycoPER), basal and compensatory glycolytic rates of CD4^+^ T cells in the four groups (control cells, SLC2A1 overexpression cells, IL‐37 treated cells, and IL‐37 treated SLC2A1 overexpression cells) were measured (*n* = 3). (I–K) Glucose uptake, lactate production and ATP production of CD4^+^ T cells in the four groups (control cells, SLC2A1 overexpression cells, IL‐37 treated cells, and IL‐37 treated SLC2A1 overexpression cells) were measured (*n* = 3). (L) After the addition of 2‐DG, flow cytometry was used to detect the differentiation of CD4^+^ T cells with or without IL‐37 treatment (*n* = 3). Error bars represent the mean ± SEM. *n* = 3 biologically independent experiments. ^*^ *p* < 0.05; ^**^ *p* < 0.01; ^***^ *p* < 0.001; ^****^ *p* <0.0001; p values were calculated using Student's t test or One‐way ANOVA and Two‐way ANOVA.

We analyzed single‐cell RNA sequencing data from patients with Crohn's disease, chronic rhinitis, and arthritis, as well as healthy controls, and found that SLC2A1 expression in CD4^+^ T cells differed significantly between patients and healthy individuals (Figure 6A–C). These findings suggest that SLC2A1 may be involved in CD4^+^ T cell dysfunction during inflammatory diseases. To further investigate whether IL‐37 regulates CD4^+^ T cell differentiation via SLC2A1, we overexpressed SLC2A1 in CD4^+^ T cells and analyzed subset differentiation by flow cytometry. SLC2A1 overexpression significantly altered the differentiation pattern of CD4^+^ T cell subsets, increasing the proportions of Th1 and Th17 cells while reducing the proportion of Th2 cells. rhIL‐37 treatment markedly attenuated these changes, decreasing the elevated Th1 and Th17 cell proportions and restoring Th2 cell levels. Treg differentiation remained unaffected under these conditions (Figure 6D). To further assess the role of SLC2A1 in CD4^+^ T cell differentiation, SLC2A1 was knocked down and subset differentiation was subsequently analyzed (Figure S8A). Flow cytometry showed that SLC2A1 deficiency significantly reduced Th1 and Th17 differentiation while promoting Th2 differentiation (Figure S8B). Notably, exogenous rhIL‐37 failed to further modulate T helper cell subset differentiation under SLC2A1 knockdown conditions (Figure S8B). Consistent with the overexpression results, Treg differentiation remained unchanged following either SLC2A1 knockdown or subsequent IL‐37 treatment. Collectively, these results indicate that SLC2A1 is an important downstream mediator through which IL‐37 regulates CD4^+^ T cell differentiation.

SLC2A1 is a key glucose transporter that mediates cellular glucose uptake and supports glycolysis. Consistent with the enrichment of glycolysis–gluconeogenesis pathways observed in our earlier analyses, we further examined whether IL‐37 regulates glycolytic activity via SLC2A1.rhIL‐37 was applied to control and SLC2A1‐overexpressing CD4^+^ T cells, and extracellular acidification rate (ECAR) and glycolytic parameters were assessed. SLC2A1 overexpression significantly enhanced ECAR, proton efflux, basal glycolysis, and compensatory glycolysis, confirming its role in promoting glycolytic activity. In contrast, IL‐37 markedly suppressed these glycolytic parameters and effectively reversed the metabolic enhancement induced by SLC2A1 overexpression (Figure 6E–H), indicating that IL‐37 inhibits glycolysis in CD4^+^ T cells. Consistently, SLC2A1 overexpression increased glucose uptake, whereas IL‐37 reduced glucose uptake and attenuated SLC2A1‐mediated enhancement (Figure 6I), suggesting that IL‐37 regulates glucose metabolism through SLC2A1. ATP production and lactate levels exhibited similar trends (Figure 6J,K), further supporting the role of IL‐37 in suppressing glycolytic metabolism. To further determine the requirement of SLC2A1 in IL‐37–mediated metabolic regulation, glycolytic function was assessed following siRNA‐mediated SLC2A1 knockdown. SLC2A1 deficiency significantly reduced ECAR and other glycolytic parameters (Figure S8C–F). Notably, IL‐37 treatment failed to further suppress glycolytic activity under SLC2A1‐deficient conditions (Figure S8C–F). Similarly, SLC2A1 knockdown reduced glucose uptake, ATP production, and lactate secretion, and no additional inhibitory effect of IL‐37 was observed (Figure S8G–I), indicating that IL‐37 requires SLC2A1 to exert its metabolic effects. Collectively, these results demonstrate that SLC2A1 is a critical mediator of IL‐37–regulated glycolytic metabolism in CD4^+^ T cells.

To determine whether IL‐37 regulates CD4^+^ T cell differentiation through glycolysis, we used the glycolysis inhibitor 2‐deoxyglucose (2‐DG). Treatment with 2‐DG markedly attenuated the effects of IL‐37 on T helper cell differentiation, including the downregulation of Th1 and Th17 cells and the upregulation of Th2 cells, indicating that IL‐37 regulates Th1, Th17, and Th2 differentiation, at least in part, through glycolytic modulation. In contrast, 2‐DG treatment did not affect IL‐37‐induced upregulation of Treg differentiation (Figure 6L), suggesting that the regulation of Treg cells by IL‐37 may involve glycolysis‐independent mechanisms. These findings further support a close link between metabolic regulation and CD4^+^ T cell differentiation downstream of IL‐37.

### 2.7 IL‐37 Regulates SLC2A1 Expression by Modulating the A2445 m^6^A Site in the 3′UTR

**FIGURE 7:**
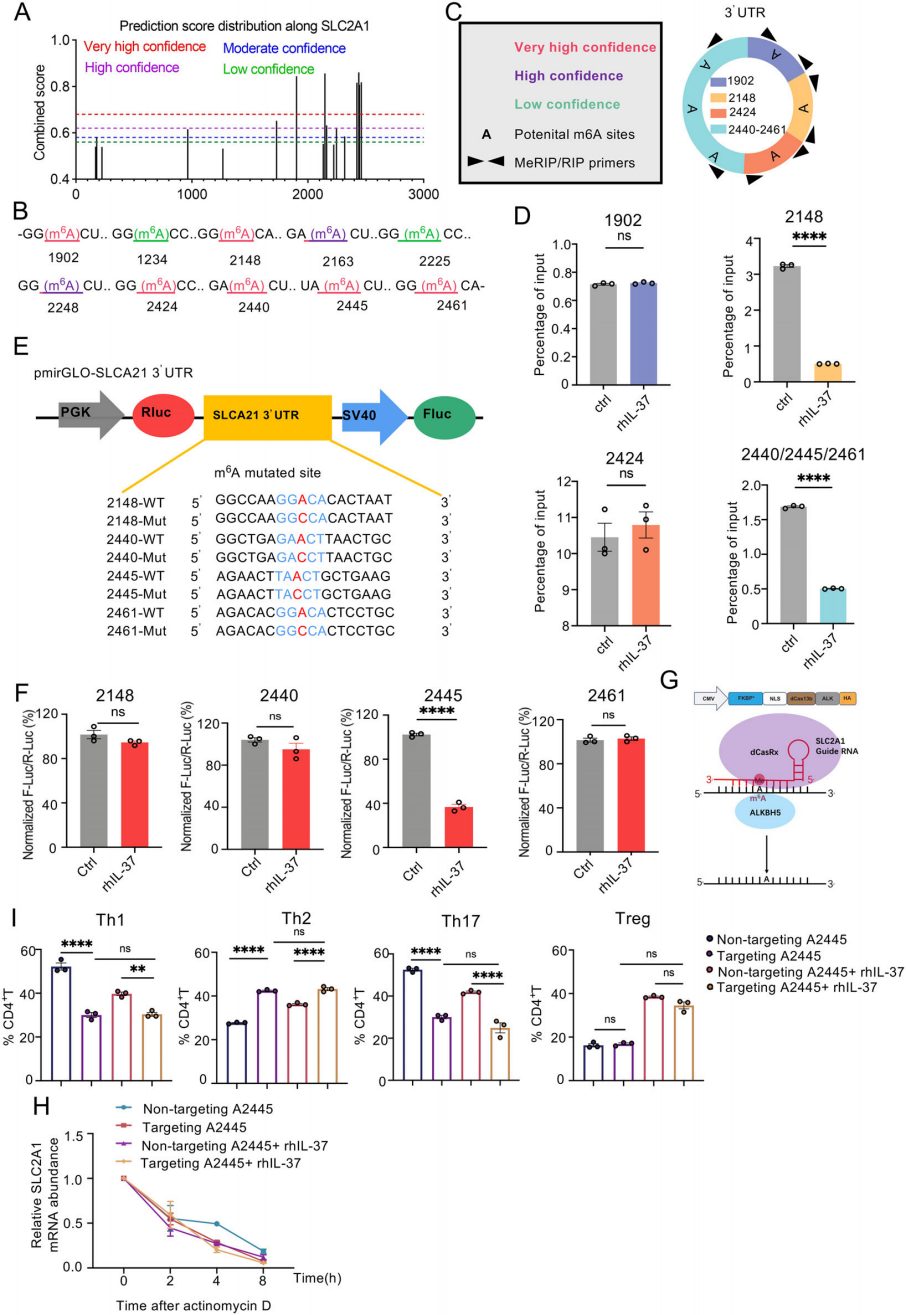
IL‐37 suppresses m^6^A modification at the A2445 site of the SLC2A1 3′UTR to reduce SLC2A1 expression. (A,B) The m^6^A sites in the 3'UTR region of SLC2A1 mRNA were predicted by the commonly used m^6^A prediction server SRAMP. (C) Schematic diagram of m^6^A IP qPCR primer design. (D) Detection of m^6^A sites regulated by IL‐37 by m^6^A IP qPCR (*n* = 3). (E) Construction of the dual‐luciferase expression vector for the m^6^A site and display of the mutant sequence. (F) Detection of the m^6^A site in the 3'UTR of SLC2A1 regulated by IL‐37 by the dual‐luciferase reporter system (*n* = 3). (G) Schematic of the CRISPR‐dCas13b system for site‐specific erasure of m^6^A modification at the A2445 site of SLC2A1 mRNA. (H) CD4^+^ T cells transfected with dCas13b‐ALKBH5 + gSLC2A1 (targeting the A2445 m^6^A site) or dCas13b‐ALKBH5 + non‐targeting gRNA (NT), with or without IL‐37 treatment, were treated with actinomycin D (5 µm). (I) Flow cytometry of Th1, Th2, Th17 and Treg cells. CD4^+^ T cells were transfected with dCas13b‐ALKBH5 + gSLC2A1 (targeting the A2445 m^6^A site) or + NT‐gRNA, with or without IL‐37 treatment, and polarized for 72 h. Error bars represent the mean ± SEM. *n* = 3 biologically independent experiments. ^*^ *p* < 0.05; ^**^ *p* < 0.01; ^***^ *p* < 0.001; ^****^ *p* <0.0001; p values were calculated using Student's t test or One‐way ANOVA and Two‐way ANOVA.

We used the SRAMP prediction server (www.cuilab.cn/m6asiteapp/old) to identify potential m^6^A sites within the SLC2A1 transcript. A total of 17 putative m^6^A sites were predicted, with all six very high‐confidence sites located in the 3′ UTR (Figure 7A,B). Based on this distribution, we focused on these high‐confidence sites for further analysis. m^6^A IP‐qPCR analysis revealed that IL‐37 significantly regulated m^6^A modification at site A2148 and within the region containing A2440/A2445/A2461 (Figure 7C,D). To determine the functional relevance of these sites, adenine residues were mutated to cytosine to generate luciferase reporter constructs (Figure 7E). Among these, mutation at A2445 resulted in a significant change in reporter activity compared with the wild‐type sequence (Figure 7F), indicating that this site plays a key role in IL‐37–mediated regulation of SLC2A1 expression. To further investigate the role of site‐specific m^6^A modification, we employed a CRISPR‐dCas13–based demethylation system to selectively remove m^6^A at the A2445 site (Figure 7G). Site‐specific m^6^A depletion markedly accelerated SLC2A1 mRNA decay, whereas IL‐37 also promoted mRNA degradation but to a lesser extent. Notably, IL‐37 failed to further enhance mRNA decay following A2445 demethylation (Figure 7H), indicating that its regulatory effect depends on this site. Flow cytometric analysis showed that removal of m^6^A at A2445 significantly reduced Th1 and Th17 differentiation while increasing Th2 differentiation, without affecting Treg cells (Figure 7I). Under these conditions, IL‐37 no longer modulated CD4^+^ T cell differentiation, further supporting the requirement of A2445 m^6^A modification. Collectively, these results indicate that IL‐37 regulates SLC2A1 expression and CD4^+^ T cell differentiation through site‐specific m^6^A modification at A2445.

To assess the potential translational relevance of our findings, we further examined whether m^6^A regulation of SLC2A1 may also be conserved in humans. Predicted m^6^A sites within the 3′ UTR of human SLC2A1 mRNA were mapped back to the gene, and single nucleotide polymorphisms (SNPs) in SLC2A1 were retrieved from the Ensembl database. Intersection analysis identified 14 SNPs located within ±2 bp of the predicted m^6^A consensus motifs (Table 1), suggesting that these variants may alter the local RRACH sequence context and thereby influence m^6^A deposition and SLC2A1 mRNA stability. Together, these results support that IL‐37 downregulates SLC2A1 expression by modulating m^6^A modification at the A2445 site within the 3′ UTR of SLC2A1 mRNA. They also suggest that IL‐37 may exert immunoregulatory effects by influencing CD4^+^ T‐cell differentiation through m^6^A‐dependent metabolic reprogramming and that this regulatory mechanism may have potential relevance in humans.

**TABLE 1:** m6A_SNP_overlap_summary.

SNP_ID	m6A_chr	m6A_pos	SNP_pos	SNP_vf	Sequence	Relative_position	snp_in_motif
**rs1643432916**	chr1	42926965	42926964	A	GCTGGATGAGACTTCCAAACC	−1	Yes
**rs1570590304**	chr1	42926965	42926965	G	GCTGGATGAGACTTCCAAACC	0	Yes
**rs1253165412**	chr1	42926881	42926879	C	AATATTCAGGACTTAACGGCT	−2	Yes
**rs1261477900**	chr1	42926848	42926849	CTT	CAAAAGCAAGACTGTTGCTCA	1	Yes
**rs1570590142**	chr1	42926708	42926706	T	GAGGGTGGAGACTAAGCCCTG	−2	Yes
**rs1325792697**	chr1	42926708	42926707	T	GAGGGTGGAGACTAAGCCCTG	−1	Yes
**rs1408556340**	chr1	42926654	42926656	G	GTAGGGCTGGACCTATGTCCT	2	Yes
**rs1044642562**	chr1	42926639	42926637	C	TGTCCTAAGGACACACTAATC	−2	Yes
**rs1482995889**	chr1	42926639	42926640	T	TGTCCTAAGGACACACTAATC	1	Yes
**rs1643429026**	chr1	42926626	42926628	—	CACTAATCGAACTATGAACTA	2	Yes
**rs543194486**	chr1	42926626	42926628	G	CACTAATCGAACTATGAACTA	2	Yes
**rs543194486**	chr1	42926626	42926628	T	CACTAATCGAACTATGAACTA	2	Yes
**rs6413524**	chr1	42926472	42926470	A	TTTACCTGAGACCAGTTGGGA	−2	Yes
**rs55728431**	chr1	42925826	42925828	A	CCTTTATTGGACAGGCTCAAA	2	Yes

To identify potential m^6^A reader proteins that recognize SLC2A1 RNA, we performed a biotinylated RNA pull‐down assay under three conditions: a negative control group (untreated cells with a scrambled biotinylated probe), a wild‐type control group (untreated cells with a target‐specific biotinylated probe), and an experimental group (rhIL‐37‐treated cells with a target‐specific biotinylated probe). Pulled‐down proteins were visualized by silver staining and identified by mass spectrometry.MS analysis showed no detectable binding in the negative control group, whereas IGF2BP1 was specifically detected in the wild‐type control group. Notably, in addition to IGF2BP1, IGF2BP2 and IGF2BP3 were also identified in the experimental group. Given that m^6^A readers are increasingly recognized as important regulators in colitis progression, including the reported role of IGF2BP2 in DSS‐induced colitis [36], these findings suggest that IGF2BP family proteins may participate in IL‐37‐mediated regulation of SLC2A1.To validate these results, we performed RIP‐qPCR to assess the binding of IGF2BP2 and IGF2BP3 to SLC2A1 RNA. In IL‐37‐treated cells, both IGF2BP2 and IGF2BP3 antibodies significantly enriched SLC2A1 RNA, whereas little or no binding was detected in control cells (Figure S9). These results suggest that IL‐37 treatment promotes the association of IGF2BP2 and IGF2BP3 with SLC2A1 RNA, supporting their potential involvement in the post‐transcriptional regulation of SLC2A1.

## 3 Discussion

Aberrant m^6^A modifications have been extensively implicated in diverse pathological processes [37, 38]. For example, in tumorigenesis, METTL3‐mediated m^6^A modification promotes the progression of Wilms’ tumor and acute myeloid leukemia (AML) by enhancing the translation of oncogenic transcripts such as MYC and EGFR [39]. In metabolic diseases, adipose tissue‐specific knockout of METTL14 disrupts UCP1 mRNA stability, driving obesity and insulin resistance [40]. Global m^6^A hypomethylation in C9ORF72‐ALS/FTD leads to transcriptome‐wide dysregulation and toxic accumulation of bidirectional repeat RNAs and dipeptide repeats (DPRs), whereas therapeutic restoration of m^6^A levels via modulation of methyltransferases or demethylases ameliorates neurodegenerative pathology [41]. Notably, immunomodulation via m^6^A editing is emerging as a frontier in inflammatory disease research: METTL3 deficiency impairs TRAF6‐mediated degradation of m^6^A ‐tagged mRNAs, amplifying NF‐κB signaling in macrophages and aggravating colitis [42]. Conversely, ALKBH5‐mediated m^6^A erasure stabilizes *Tbx21* transcripts, promoting Th1 differentiation and exacerbating experimental autoimmune encephalomyelitis (EAE) [43]. Collectively, these findings underscore the importance of dissecting m^6^A ‐related dysregulation for potential therapeutic interventions across diverse disease contexts.

Although multiple mechanistic studies suggest that IL‐37 may alleviate inflammation by modulating T‐cell subset balance [44, 45, 46], its role in regulating immunometabolic reprogramming through mRNA modification remains unclear. Here, we demonstrate that IL‐37 functions as an m^6^A methylation inhibitor to modulate CD4^+^ T cell differentiation and suppress adoptive transfer colitis. Mechanistically, IL‐37 downregulates METTL14 expression via SIGIRR‐mediated inhibition of NF‐κB P65 signaling, resulting in reduced m^6^A abundance in CD4^+^ T cells and altered T cell differentiation. In adoptive transfer colitis exacerbated by METTL14 overexpression in CD4^+^ T cells, IL‐37 effectively reversed disease severity. Concurrently, we identified that IL‐37 regulates m^6^A deposition on numerous metabolic genes, with glycolysis‐related transcripts being predominantly affected. Notably, IL‐37 suppresses CD4^+^ T cell glycolysis by destabilizing SLC2A1 mRNA through the A2445 m^6^A site in its 3′UTR, leading to downregulation of SLC2A1 and subsequent modulation of Th1/Th17 vs. Th2 differentiation. These findings reveal a novel anti‐inflammatory mechanism in which IL‐37 orchestrates metabolic reprogramming and T cell differentiation through m^6^A methylation.

As a pivotal epitranscriptomic mechanism, m^6^A RNA modification integrates transcriptional plasticity with post‐transcriptional regulation, fundamentally shaping immune cell differentiation, inflammatory responses, and anti‐tumor immunity [47, 48, 49]. Recent studies indicate that METTL3‐mediated m^6^A methylation stabilizes *Foxp3* mRNA, enhancing Treg immunosuppressive function [50, 51]. The deletion of FTO enhances the anti‐tumor activity of CD8^+^ T cells by stabilizing IFN‐γ transcripts [51, 52]. These findings reveal the dual role of m^6^A in balancing immune tolerance and activation. In innate immunity, m^6^A modification precisely regulates macrophage polarization and dendritic cell maturation. A study in 2023 demonstrated that the m^6^A reading protein YTHDF1 can enhance the translation of Nlrp3 mRNA in LPS‐pretreated macrophages, promote the activation of NLRP3 inflammasome, and exacerbate sepsis‐related inflammation [53]. Conversely, the deletion of m^6^A in DC can disrupt the stability of MHC‐I transcripts and impair the ability of antigen cross‐presentation, suggesting that it is indispensable for the initiation of adaptive immunity [54]. Additionally, tumors exploit m^6^A ‐mediated PD‐L1 mRNA stability to evade immune surveillance, while small molecule inhibitors targeting FTO can reverse this effect and synergize with anti‐PD‐1 therapy [55]. The interaction between m^6^A and immune checkpoint regulation further highlights its therapeutic significance. Therefore, revealing the mechanism by which m^6^A regulates cell differentiation and disease occurrence is of great significance for new strategies in disease immunotherapy.

In adaptive immunity, the differentiation of CD4^+^ T cells are tightly linked to the m^6^A ‐glycolysis axis. METTL14 promotes glycolytic Th17 activity and IL‐17 secretion via m^6^A ‐stabilized *HIF‐1α* mRNA, exacerbating autoimmune pathology [56]. YTHDF2 promotes the immunosuppressive function of regulatory T cells by recognizing the m^6^A site of PDK4 mRNA, accelerating its degradation and inhibiting glycolysis [57]. In obesity models, adipose‐specific knockout of ALKBH5 improves insulin sensitivity by increasing m^6^A methylation of PGC‐1α mRNA, suppressing glycolysis, and enhancing oxidative phosphorylation [58]. In rheumatoid arthritis, METTL3 upregulates ENO1 in an m^6^A ‐dependent manner, promoting glycolytic activity in synovial fibroblasts and inflammatory factor production [59]. Our findings extend these observations, showing that IL‐37 modulates glycolysis in CD4^+^ T cells via METTL14‐dependent destabilization of SLC2A1, thereby fine‐tuning Th cell differentiation.

In the present study, we investigated the protective role of IL‐37 in alleviating colitis through suppression of METTL14. Our findings showed that downregulation of METTL14 reduced m^6^A enrichment on SLC2A1 mRNA, thereby attenuating glycolysis and suppressing inflammation. However, it should be noted that the role of METTL14 in inflammatory regulation remains controversial, as existing studies have reported somewhat inconsistent findings. Some studies support a pro‐inflammatory consequence of METTL14 downregulation. For example, METTL14 expression is markedly decreased during the inflammation‐to‐cancer transition in colorectal cancer, and reduced METTL14 expression is associated with poor overall survival in patients. Moreover, METTL14 overexpression has been shown to inhibit the migration, invasion, and metastasis of colorectal cancer cells [60]. Similarly, METTL14 expression is significantly reduced in the livers of patients with MAFLD and in multiple mouse models, whereas hepatocyte‐specific knockdown of this methyltransferase aggravates lipid accumulation, liver injury, and fibrosis. In contrast, hepatic overexpression of METTL14 in high‐fat diet‐fed mice alleviates these pathophysiological changes [61]. On the other hand, a substantial number of studies have also reported an anti‐inflammatory effect of METTL14 downregulation, which is consistent with our findings. For instance, knockdown of METTL14 has been shown to ameliorate cellular senescence, inflammatory responses, and oxidative stress in senescent endothelial cells [62]. Likewise, deletion of METTL14 in astrocytes significantly affects the expression of the negative inflammatory regulator DUSP1, thereby attenuating MAPK signaling [63]. We speculate that this seemingly paradoxical dual role of METTL14 in inflammation may arise from several key factors. First, the function of METTL14 is likely to be highly dependent on the cellular context in which it operates. In different cell types, METTL14 may interact with distinct signaling molecules or receptors, leading to divergent biological outcomes. Second, inflammation is a dynamic process, and the role of METTL14 may vary across different stages of inflammation or under specific pathological conditions. Different studies may therefore capture distinct phases of the inflammatory response. In addition, differences in experimental approaches, animal strains, or cellular origins may also partially account for the heterogeneity among studies. Taken together, these observations suggest that METTL14 exerts a complex and potentially context‐dependent regulatory role in inflammation. Future studies are needed to further elucidate the key molecular switches that determine whether METTL14 functions in a pro‐inflammatory or anti‐inflammatory manner, to define its specific upstream and downstream signaling networks, and to clarify its dynamic regulation at different stages of diseases. Elucidating these condition‐dependent mechanisms will be essential for the precise therapeutic targeting of METTL14 in inflammatory disorders.

Although this study has elucidated the role of the IL‐37–m^6^A–SLC2A1 axis in T‐cell regulation, several limitations warrant further discussion. First, while we confirmed that METTL14 exerts regulatory effects through SLC2A1, its potential influence on other substrate genes and the functional contributions of m^6^A reader proteins IGF2BP2 and IGF2BP3 remain incompletely understood. These aspects require further clarification through systematic approaches such as transcriptome‐wide m^6^A sequencing, CLIP‐seq, and mechanistic studies combining gene knockdown and rescue experiments. Second, although we validated the overexpression patterns of METTL14 and SLC2A1 in human CD4^+^ T cells, the clinical translational value of IL‐37 still needs to be assessed in well‐stratified cohorts of inflammatory bowel disease (IBD) patients, supplemented by humanized mouse models or intestinal organoid testing. Third, while this study primarily focused on glucose metabolism pathways, potential interactions with oxidative phosphorylation, fatty acid metabolism, and glutaminolysis may have been overlooked. Follow‐up studies should integrate metabolomics and isotope tracing techniques to comprehensively map the metabolic network regulated by this signaling axis. Lastly, although the experimental data from mouse colitis models were robust, the development of potential therapeutic strategies—such as METTL14 inhibitors, GLUT1 modulators, or recombinant IL‐37—still requires systematic preclinical evaluation of safety and efficacy. Further validation using human samples, such as primary intestinal lamina propria T cells from IBD patients, is also essential.

In this study, the cascade pathway “IL‐37‐SIGIRR‐METTL14‐ m^6^A ‐SLC2A1” was proposed as the core axis of regulating T cell metabolism and differentiation, which not only expanded the theoretical framework of IL‐37 immunosuppression mechanism. It also provides a new metabolic epigenetic combined intervention strategy for the treatment of Th1/ Th17‐driven inflammatory diseases such as IBD.

## 4 Methods

### 4.1 Cell Lines

Human embryonic kidney 293T (HEK293T) cells (Homo sapiens; female;RRID: CVCL_0063) were purchased from the American Type Culture Collection (ATCC, CRL‐3216, Manassas, VA, USA). Cells were cultured in Dulbecco's modified Eagle's medium (DMEM; Thermo Fisher Scientific) supplemented with 10% fetal bovine serum (FBS) and 1% penicillin/streptomycin at 37 °C in a humidified atmosphere containing 5% CO_2_. All cells were found to be free from mycoplasma contamination.

### 4.2 T‐Cell Isolation and Culture

Naïve CD4^+^ T cells were isolated from mouse spleens using the Naïve CD4^+^ T Cell Isolation Kit (19 765, STEMCELL Technologies) following the manufacturer's protocol. Cells were cultured in RPMI‐1640 medium supplemented with 10% fetal bovine serum (FBS), 1% penicillin–streptomycin, 50 µm β‐mercaptoethanol, 1 × non‐essential amino acids(11140‐050, Gibco), and 1 × sodium pyruvate(11136‐070, Gibco) [64].

### 4.3 CD4^+^T Cell Proliferation and Differentiation

Naïve CD4^+^ T cells were labeled with 5 µm CFSE (21888, Sigma–Aldrich) for 5 min at 37 °C, quenched with complete RPMI‐1640 medium, washed, and resuspended at the desired concentration. Cells were stimulated with anti‐CD3/CD28 magnetic beads (11452D, Thermo Fisher Scientific,2µL/100µL culture medium) and cultured under lineage‐specific polarizing conditions. Th1 differentiation was induced with 10 ng/mL IL‐2(212‐12,PeproTech), 1 ng/mL IL‐12 (210‐12, PeproTech), and 10 µg/mL anti‐IL‐4 (BE0045, BioXcell); Th2 with 10 ng/mL IL‐2, 20 ng/mL IL‐4 (214‐14, PeproTech), and 20 µg/mL anti‐IFN‐γ (BE0055, BioXcell); Th17 with 20 ng/mL IL‐6 (216‐16, PeproTech), 10 ng/mL IL‐23 (CT028‐M08H, Sino Biological), 2 ng/mL TGF‐β (100‐21, Sino Biological), anti‐IL‐4 (10 µg/mL), and anti‐IFN‐γ (10 µg/mL); and Treg with IL‐2 (10 ng/mL), TGF‐β (5 ng/mL), anti‐IL‐4 (10 µg/mL), and anti‐IFN‐γ (10 µg/mL). All cultures were maintained in complete RPMI‐1640 medium at 37 °C in 5% CO_2_ [65]. Both proliferation and differentiation were measured after 72 h of cell culture.

### 4.4 Dot Blot

RNA samples were denatured at 65 °C for 5 min, chilled on ice, and diluted to equal volumes. Aliquots were spotted onto nitrocellulose membranes, air‐dried, and UV cross‐linked. Membranes were blocked with 5% BSA in TBST for 1 h and incubated with anti‐m^6^A antibody (ab151230, Abcam,1:1000) for 1 h at room temperature. After three washes with TBST, membranes were incubated with the appropriate secondary antibody for 2 h, followed by additional washes before detection [66, 67].

### 4.5 Quantitative Real‐Time PCR & m^6^A IP RT‐qPCR

Total RNA was extracted using TRIzol reagent (Thermo Fisher Scientific) and assessed by capillary electrophoresis (NanoDrop 2000; Thermo Fisher Scientific). cDNA was synthesized with the PrimeScript RT reagent kit with gDNA Eraser (Takara Bio). qPCR was performed in triplicate using gene‐specific primers (Qing Ke Bio; sequences in Table 2) and TB Green Premix Ex Taq II (Takara Bio) on a LightCycler96 system (Roche). β‐actin served as the internal control, and relative expression was calculated using the 2^‐ΔΔCt method. m^6^A IP RT‐qPCR was conducted with the MeRIP RT‐qPCR kit (IVDSHOW, AP‐9028) following the manufacturer's instructions.

**TABLE 2:** Primer sequence.

SLC2A1‐F	GATCCCAGCAGCAAGAAGGT
SLC2A1‐R	GAGAGACCAAAGCGTGGTGA
PFKP‐R	TTGGGACAAAACGCACCCTA
PFKP‐F	CGTTGCTAATAGTGGCAGGC
1902‐F	CCTTTCCTCAGCCAGCAGTG
1902‐R	GAATAGATCTGAGCAGCAGTC
2148‐F	CTGTCCAGACACTTGCCTTC
2148‐R	GTGGCAGCTACCGCCTC
2424‐F	AGAGGGAAGGGCCAGGCTG
2424‐R	TCAGCCTCCGAGGTCCTTCTC
2440‐2445‐2461‐F	GGCTGAGAACTTAACTG
2440‐2445‐2461‐R	GGTGTATTTACAAGTGGGTTTG
METTL3‐F	AGCCTTCTGAACCAACAGTCC
METTL3‐R	CCGACCTCGAGAGCGAAAT
METTL14‐F	TTTCTCTGGTGTGGTTCTGG
METTL14‐R	AAGTCTTAGTCTTCCCAGGATTG
WTAP‐F	CTAGCAACCAAAGAGCAGGA
WTAP‐R	CTGGCAAATGGACCAAGTAATG
FTO‐F	GTCAGAGAGAAGGCCAATGAA
FTO‐R	CTCTGCTCTTAAGGTCCACTTC
ALKBH5‐F	ACAAGATTAGATGCACCGCG
ALKBH5‐R	TGTCCATTTCCAGGATCCGG
GAPDH‐F	AGGTCGGTGTGAACGGATTTG
GAPDH‐R	TGTAGACCATGTAGTTGAGGTCA

### 4.6 Western Blot

Proteins were extracted with RIPA buffer (Beyotime) containing protease/phosphatase inhibitors (Bimake), quantified by BCA assay, separated by SDS‐PAGE, and transferred to PVDF membranes (Millipore). Membranes were incubated with primary antibodies overnight at 4 °C and secondary antibodies for 1 h at room temperature. Signals were detected using ECL (Thermo Fisher) and visualized on a ChemiDoc XRS+ system (Bio‐Rad); antibodies included GAPDH (#2118, CST, 1:1000), METTL3 (ab195352, Abcam, 1:1000), METTL14 (ab219722, Abcam, 1:1000), FTO (#13441, CST, 1:1000), ALKBH5 (67811‐1‐Ig, Proteintech, 1:2000), WTAP (60188‐1‐Ig, Proteintech, 1:2000), and SLC2A1 (66290‐1‐Ig, Proteintech, 1:1000).

### 4.7 Lentivirus or Adenovirus Infection

Naïve CD4^+^ T cells were stimulated with anti‐CD3/CD28 for 24 h and transduced with lentivirus (MOI = 100) or adenovirus (MOI = 1000)(Hanbio Tech). Cells were cultured at 37 °C with 5% CO_2_, and medium was replaced after 10–16 h. If the infection efficiency is low, perform a second infection after 24 h to improve the efficiency.

### 4.8 Inhibitor Treatment of CD4^+^ T Cells

Naïve CD4^+^ T cells were activated with anti‐CD3/CD28 antibodies (16‐0031‐82,16‐0281‐82, eBioscience, anti‐CD3 2ug/mL, anti‐ CD28 1ug/mL) for 24 h and seeded into 24‐well plates. The inhibitor (S8531, S1460, S7351, S9876, Selleck) was dissolved in DMSO (Thermo Fisher Scientific), diluted in medium, and applied at a final concentration of 5 µm. Cells were cultured at 37 °C with 5% CO_2_. Cells were harvested after 72 h of inhibitor treatment.

### 4.9 siRNA Interferes With CD4^+^ T Cell Gene Expression

For siRNA transfection, 2.5 µL of 20 µm siRNA duplex (Dharmacon) was diluted in 100 µL serum‐free medium Opti‐MEM (31985062, Thermo Fisher Scientific) and mixed gently. RNAFit reagent (5 µL) was added, vortexed briefly, and incubated at room temperature for 10 min to form complexes. The transfection mixture was then added to 150 µL of complete medium in the target well. Cells were harvested after 72 h of inhibitor treatment.

### 4.10 Mouse Colon Tissue HE

Mouse colons were fixed in 4% paraformaldehyde, paraffin‐embedded, and sectioned at 5 µm. Sections were stained with hematoxylin and eosin and imaged using an Olympus BX600 microscope with a SPOT Flex camera. Colonic inflammation was scored according to the criteria described in Table 3. Histological scoring was performed by an investigator blinded to group allocation and treatment.

**TABLE 3:** Pathological scoring criteria for colitis.

Scoring items	Scoring criteria examples
Inflammation severity	0 = None; 1 = Mild; 2 = Moderate; 3 = Severe
Inflammation extent	0 = None; 1 = Mucosa; 2 = Mucosa + submucosa; 3 = Full ‐ thickness inflammation
Crypt damage	0 = No damage; 1 = Loss of a small number of goblet cells; 2 = Partial destruction of tubular glands; 3 = Complete loss of tubular glands
Epithelial erosion	0 = None; 1 = Focal; 2 = Regional; 3 = Widespread
Edema	0 = None; 1 = Mild; 2 = Moderate; 3 = Severe |

### 4.11 Mouse Colon Tissue Flow Cytometry

Mouse colons were minced, and the resulting cell suspension was filtered through a 70 µm strainer, centrifuged, and resuspended for flow cytometric analysis. Cells were stained with the following antibodies: CD3‐PE‐CY7(100220, BioLegend), CD4‐APC‐Fire 750 (100567, BioLegend), IFN‐γ‐FITC (163512, BioLegend), IL‐17‐APC (506916, BioLegend), IL‐6‐APC (504508, BioLegend), TNF‐a‐APC‐CY7 (12117, BioLegend), IL‐10‐FITC (505006, BioLegend).

### 4.12 Dual‐Luciferase Reporter System Detects m^6^A Sites and Transcription Factors

The plasmid transfection ratio is DNA: Lipofectamine 293T = 1 µg: 2.5 µL. 48 h after transfection, the Dual‐Glo Luciferase Assay System kit (E2920, Promega) was used to detect the activities of Renilla and Luciferase luciferases.

### 4.13 Glycolysis, Glucose Consumption, Lactate Production and ATP Production

Glycolytic activity of CD4^+^ T cells was assessed using the Seahorse XF Glycolytic Rate Assay Kit (103344‐100, Agilent) following the manufacturer's instructions. Glucose uptake, lactate production, and ATP levels were measured using colorimetric assay kits (S0202S, S0208S, S0026, Beyotime Biotechnology) according to the manufacturer's protocols.

### 4.14 Assessment of m^6^A Abundance

Quantitative analysis of m^6^A abundance ELISA and Mouse serum ELISA analysis detection was performed according to the instructions of Epigentek EpiQuik m^6^A RNA (p‐9005‐96) Methylation Quantification Kit. According to the instructions of the purchased kit, the main experimental steps are as follows: Prepare standards, dilute serum, incubate at 37°C for 90 min, wash, antibody bind, wash, and develop color. The following kits were used for the detection of relevant inflammatory factors in mouse serum: RX203075M, RX203097M, RX203049M, RX202449M, RX202402M, RX203066M (Ruixin Biological).

### 4.15 Sequencing Sample Procession

CD4^+^ T cells treated with PBS or rhIL‐37 (7585‐IL‐025, R&D Systems) for 72 h were washed three times with RNase‐free PBS. RNA was extracted using TRIzol, and quality and concentration were assessed by NanoDrop 2000 and agarose gel electrophoresis. Qualified RNA samples were submitted to Novogene for RNA‐seq and m^6^A MeRIP‐seq.

### 4.16 Animal Model

The IL‐37tg mice and Rag2^−/−^ mice were purchased from Cyagen Biosciences. All animal experiments were conducted in accordance with national and local guidelines and were approved by the Institutional Animal Care and Use Committee of State Key Laboratory of Biotherapy (Approval No.20230307051). Procedures were performed following the principles of animal welfare, minimizing animal numbers and alleviating pain and distress. Animals were housed under specific pathogen‐free (SPF) conditions.

### 4.17 Adoptive Transfer Colitis Model Description

CD4^+^CD45RB^hi^ T cells were isolated from murine spleens and activated in vitro through stimulation with anti‐CD3 and anti‐CD28 antibodies. Following activation, the T cells were subjected to various experimental interventions, including treatment with recombinant IL‐37 protein or lentiviral‐mediated gene transduction, and subsequently transferred into immunodeficient Rag2^−/−^ mice via tail vein injection to induce colitis. Animals were randomly assigned to blocks by baseline body weight prior to the experiment. For blinding, drug solutions were prepared and coded by an independent technician, and administered by a second technician using syringes labeled only with animal IDs. Outcome assessments were conducted by researchers blinded to group assignment. The coding technician performed unblinding after data collection for statistical analysis.

### 4.18 Targeted m^6^A Erasure Using Cas13d‐ALKBH5 Fusion System

The dCasRx‐ALKBH5 plasmid (Addgene plasmid #175582), encoding NLS‐dRfxCas13d‐NLS‐ALKBH5. gRNA targeting the A2445 site within the 3′ UTR of SLC2A1 mRNA was designed, and the sequence of the resulting targeting construct is as follows:

U6‐dCasRX grna(NM_001424864.1 c.2445)‐1

GAGGGCCTATTTCCCATGATTCCTTCATATTTGCATATACGATACAAGGCTGTTAGAGAGATAATTAGAATTAATTTGACTGTAAACACAAAGATATTAGTACAAAATACGTGACGTAGAAAGTAATAATTTCTTGGGTAGTTTGCAGTTTTAAAATTATGTTTTAAAATGGACTATCATATGCTTACCGTAACTTGAAAGTATTTCGATTTCTTGGCTTTATATATCTTGTGGAAAGGACGAAAAAACACCGAACCCCTACCAACTGGTCGGGGTTTGAAACCAGCAGTTAAGTTCTCAGCCTCCTTTTTTTT

The above sequence was inserted into the dCasRx‐ALKBH5 plasmid through the NdeI and MluI restriction sites [68, 69].

### 4.19 Statistical Analysis

All statistical analyses were performed using GraphPad Prism 8.0 software. Initially, the Shapiro–Wilk test was employed to assess whether the data followed a normal distribution. For comparisons between two groups, a two‐tailed independent samples t‐test was applied when the data were normally distributed and exhibited homogeneity of variance; otherwise, the Mann–Whitney U test was used. For comparisons involving three or more groups, one‐way or two‐way analysis of variance (ANOVA) was conducted, followed by post‐hoc multiple comparison tests such as Tukey or Bonferroni corrections as appropriate. All the experimental data were expressed as mean ± standard error. Statistical significance is indicated as ^*^ *p* < 0.05, ^**^ *p* < 0.01, ^***^ *p* < 0.001, ^****^ *p* < 0.0001; ns denotes no significance.

## Author Contributions

Conceptualized: Xiaoyan Wang and Jiong Li, Methodology: Xiaoyan Wang and Jiong Li, Investigation: Xiaoyan Wang, Jiadong Yu, Guolin Li,Hong Zhou, Pei Zhou, Jing Hu, Yifan Zhou, Linna Gu, Ya Li, Yuting Feng, Fanlian Zeng, Fulei Zhao, Qixiang Zhao, Chen Zhang, Mingxiang He, Wenlin Wu, Xinai Cui, Nongyu Huang, Shishi Huang, Xinai Cui, and Jiong Li, Visualization: Wenling Wu, Kaijun Cui and Nongyu Huang, Writing – original draft: Xiaoyan Wang, Writing – review & editing: Xiaoyan Wang, Jiadong Yu, Guolin Li and Jiong Li.

## Conflicts of Interest

The authors declare no conflicts of interest.

## Supporting information




**Supporting File**: advs75939‐Sup‐0001‐Figure S1‐S9.docx.

## Data Availability

The data that support the findings of this study are available from the corresponding author upon reasonable request.
