# Supplementary material for: m6A‐Mediated Glycolysis by IL‐37 Drives T Cell Metabolic Reprogramming to Regulate Colitis
Source: Adv Sci (Weinh). 2026 Jun 9;13(49):e20472. doi: 10.1002/advs.202520472 (PMC13336486; doi:10.1002/advs.202520472)
Supplement: Supplementary file 1 [file ADVS-13-e20472-s001.docx]

**sFigure1. Expression of m^6^A methylation key enzymes in different human immune cells.** (A) Expression of METTL3 in different Human immune cells in The Human Protein Atlas database. (B) Expression of WTAP in different Human immune cells in The Human Protein Atlas database. (C) Expression of ALKBH5 in different Human immune cells in The Human Protein Atlas database.

**sFigure2. IL-37 regulates the differentiation of CD4^+^T cells but has no effect on their proliferation and apoptosis.** (A) Flow cytometry was used to detect the differentiation of effector subsets of CD4^+^T cells with or without IL-37. (B) Statistical chart of the proportion of different effector subsets of CD4^+^T cell differentiation in CD4^+^T cells with or without IL-37 intervention (n=3). (C) Proliferation of CFSE-labeled CD4^+^T cells under the action of Anti-CD3/CD28 microspheres for 72 hours with or without IL-37 intervention. (D) Percentage of proliferating cells statistical chart (n=5). (E) Detect the apoptosis of CD4^+^T cells cultured in vitro with Anti-CD3/CD28 beads for 72 hours using Annexin V and 7AAD with or without IL-37 intervention. (F) Percentage of apoptotic cells statistical chart (n=5). Error bars represent the mean ± SEM. n=3 biologically independent experiments. ns, not significant; ***p < 0.001; ****P<0.0001; p values were calculated using Student’s t test.

**sFigure3. IL-37 inhibits the occurrence and development of adoptive transfer colitis.** (A) Schematic diagram of the adoptive transfer colitis model in Rag2^-/-^ mice. (B) Changes in body weight of mice in the control group and the rhIL-37 treatment group during the development of adoptive transfer colitis (n=6). (C) Disease severity scores of mice in the control group and the rhIL-37 treatment group during the development of adoptive transfer colitis (n=6). (D) HE staining of colon tissues from mice in the control group and the IL-37 treatment group 8 weeks after adoptive transfer colitis modeling (scale bar =200 μm or 50 μm). (E) Colon length of mice in the control group and the IL-37 treatment group 8 weeks after adoptive transfer colitis modeling. (F) Statistical chart of colon length in mice (n=6). (G) Spleen size of mice in the control group and the IL-37 treatment group 8 weeks after adoptive transfer colitis modeling. (H) Schematic diagram of mesenteric lymph node size in mice in the control group and the IL-37 treatment group 8 weeks after adoptive transfer colitis modeling. (I) RT-qPCR detection of mRNA expression of inflammatory factors IFN-γ, IL-10, IL-17A, IL-6 and TNF-α in colon tissues of mice in the control group and the IL-37 treatment group (n=6). (J) ELISA detection of protein levels of IFN-γ, TNF-α, IL-6, IL-17A and IL-10 in serum of mice in the control group and the IL-37 treatment group (n=6). Error bars represent the mean ± SEM. n = 6 biologically independent mice per group. *p < 0.05; **p < 0.01; ***p < 0.001; **** p <0.0001; p values were calculated using Student’s t test or Two-way ANOVA.

**sFigure4. IL-37 has no effect on the abundance of m^6^A methylation in naive CD4^+^T cells.** (A) Cellular immunofluorescence was used to detect the effect of IL-37 on the m^6^A methylation abundance of immature CD4^+^T cells. (B) Statistical graph of cell fluorescence intensity (n=3). (C) The effect of IL-37 on the m^6^A methylation abundance of immature CD4^+^T cells was detected by m^6^A ELISA quantitative analysis (n=3). Error bars represent the mean ± SEM. n=3 biologically independent experiments. ns, not significant; p values were calculated using Student’s t test.

**sFigure5.** **CD4⁺ T-cell differentiation following METTL14 knockdown.** (A)The expression levels of METTL14 protein in cells transfected with si-NC (negative control) and si-METTL14 were detected by Western blot. (B) Flow cytometry shows that IL-37 can counteract the differentiation shift of CD4^+^ T cell subsets Th1, Th2, Th17, and Treg caused by METTL14 knockdown (n=3). Error bars represent the mean ± SEM. n=3 biologically independent experiments. ns, not significant; *p < 0.05; **p < 0.01; ***p < 0.001; **** p <0.0001; p values were calculated using Student’s t test.

**sFigure6.** **FTO does not contribute to the differentiation of CD4^+^ T cells.** (A) RT-qPCR was used to detect the expression of FTO in CD4^+^ T cells treated with solvent and Dac51 (n=3). (B) Dot blot hybridization was used to detect the m6A methylation abundance in CD4^+^ T cells treated with solvent and Dac51. (C) Flow cytometry was used to evaluate the effect of Dac51-mediated inhibition of FTO expression on the differentiation of CD4⁺ T cell subsets, including Th1, Th2, Th17, and Treg cells (n = 3). (D) The expression levels of FTO protein in cells transfected with si-NC (negative control) and si-FTO were detected by Western blot. (E) Flow cytometry was used to evaluate the effect of si-FTO-mediated knockdown of FTO expression on the differentiation of CD4⁺ T cell subsets, including Th1, Th2, Th17, and Treg cells (n = 3). Error bars represent the mean ± SEM. n=3 biologically independent experiments. ns, not significant; *p < 0.05; **p < 0.01; ***p < 0.001; **** p <0.0001; p values were calculated using Student’s t test or One-way ANOVA and Two-way ANOVA.

**sFigure7. the expression profiles of METTL14 and GLUT1 in the DSS model.** (A)Immunofluorescence analysis of CD4⁺T cells and METTL14 expression in intestinal tissues from WT and IL-37tg mice with DSS-induced colitis. (B)Immunofluorescence analysis of CD4⁺T cells and GLUT1 expression in intestinal tissues from WT and IL-37tg mice with DSS-induced colitis.

**sFigure8.** **SLC2A1 knockdown attenuates glycolysis.** (A) The expression levels of GLUT1 protein in cells transfected with si-NC (negative control) and si-SLC2A1 were detected by Western blot. (B) Flow cytometry was used to detect the differentiation of CD4⁺ T cells in four groups (control cells, SLC2A1 knockdown cells, IL-37 treated cells, and IL-37 treated SLC2A1 knockdown cells) (n=3). (C-F) ECAR, glycolytic capacity (glycol PER), basal and compensatory glycolytic rates of CD4⁺ T cells in the four groups (control cells, SLC2A1 knockdown cells, IL-37 treated cells, and IL-37 treated SLC2A1 knockdown cells) were measured (n=3). (G-I) Glucose uptake, lactate production and ATP production of CD4⁺ T cells in the four groups (control cells, SLC2A1 knockdown cells, IL-37 treated cells, and IL-37 treated SLC2A1 knockdown cells) were measured (n=3). Error bars represent the mean ± SEM. n=3 biologically independent experiments. ns, not significant; *p < 0.05; **p < 0.01; ***p < 0.001; **** p <0.0001; p values were calculated using Student’s t test or One-way ANOVA and Two-way ANOVA.

**sFigure9.** **The m^6^A reader proteins binding to the SLC2A1 A2445 m^6^A site**. RIP assays were performed using anti-IGF2BP2 and anti-IGF2BP3 antibodies, followed by qPCR analysis of SLC2A1 mRNA enrichment (n = 3). Error bars represent the mean ± SEM. n=3 biologically independent experiments. ns, not significant; **** p <0.0001; p values were calculated using Student’s t test.
